# Synergy of Venetoclax and 8-Chloro-Adenosine in AML: The Interplay of rRNA Inhibition and Fatty Acid Metabolism

**DOI:** 10.3390/cancers14061446

**Published:** 2022-03-11

**Authors:** Dinh Hoa Hoang, Corey Morales, Ivan Rodriguez Rodriguez, Melissa Valerio, Jiamin Guo, Min-Hsuan Chen, Xiwei Wu, David Horne, Varsha Gandhi, Lisa S. Chen, Bin Zhang, Vinod Pullarkat, Steven T. Rosen, Guido Marcucci, Ralf Buettner, Le Xuan Truong Nguyen

**Affiliations:** 1 Gehr Family Center for Leukemia Research, City of Hope National Medical Center, Hematology Malignancies and Stem Cell Transplantation Institute, Duarte, CA 91010, USA; hhoang@coh.org (D.H.H.); coreymorales4@gmail.com (C.M.); irodriguezrodri@coh.org (I.R.R.); mvalerio@coh.org (M.V.); jguo@coh.org (J.G.); bzhang@coh.org (B.Z.); vpullarkat@coh.org (V.P.); srosen@coh.org (S.T.R.); gmarcucci@coh.org (G.M.); 2 Irell & Manella Graduate School of Biological Sciences, City of Hope National Medical Center, Duarte, CA 91010, USA; 3 City of Hope National Medical Center, Integrative Genomics Core, Beckman Research Institute, Duarte, CA 91010, USA; minchen@coh.org (M.-H.C.); xwu@coh.org (X.W.); 4 Department of Molecular Medicine, City of Hope National Medical Center, Duarte, CA 91010, USA; dhorne@coh.org; 5 Department of Experimental Therapeutics, The University of Texas MD Anderson Cancer Center, Houston, TX 77030, USA; vgandhi@mdanderson.org (V.G.); lschen@mdanderson.org (L.S.C.)

**Keywords:** acute myeloid leukemia, venetoclax, 8-chloro-adenosine, rRNA synthesis, metabolism

## Abstract

**Simple Summary:**

Treatment failures of acute myeloid leukemia (AML) have been attributed to the persistence of leukemia stem cells (LSCs), which are refractory to conventional treatments. Venetoclax (VEN), currently FDA-approved in combination with low-dose cytarabine or hypomethylating agents, is highly effective in inducing disease remission in patients with de novo AML; however, most of these patients eventually relapse; thus, novel VEN combinations are urgently needed. In this regard, 8-chloro-adenosine (8-Cl-Ado) is a novel RNA-directed nucleoside analog that targets AML cells, including LSCs. We demonstrate that VEN and 8-Cl-Ado cooperate in targeting ribosomal RNA synthesis and mitochondrial metabolism in LSCs, thereby decreasing LSC survival. Given the emerging concept that LSC behavior is strongly associated with protein synthesis regulation and mitochondrial metabolism, our results suggest that the VEN/8-Cl-Ado combination is a promising regimen for the treatment of patients with relapsed AML.

**Abstract:**

It is known that 8-chloro-adenosine (8-Cl-Ado) is a novel RNA-directed nucleoside analog that targets leukemic stem cells (LSCs). In a phase I clinical trial with 8-Cl-Ado in patients with refractory or relapsed (R/R) AML, we observed encouraging but short-lived clinical responses, likely due to intrinsic mechanisms of LSC resistance. LSC homeostasis depends on amino acid-driven and/or fatty acid oxidation (FAO)-driven oxidative phosphorylation (OXPHOS) for survival. We recently reported that 8-Cl-Ado and the BCL-2-selective inhibitor venetoclax (VEN) synergistically inhibit FAO and OXPHOS in LSCs, thereby suppressing acute myeloid leukemia (AML) growth in vitro and in vivo. Herein, we report that 8-Cl-Ado inhibits ribosomal RNA (rRNA) synthesis through the downregulation of transcription initiation factor TIF-IA that is associated with increasing levels of p53. Paradoxically, 8-Cl-Ado-induced p53 increased FAO and OXPHOS, thereby self-limiting the activity of 8-Cl-Ado on LSCs. Since VEN inhibits amino acid-driven OXPHOS, the addition of VEN significantly enhanced the activity of 8-Cl-Ado by counteracting the self-limiting effect of p53 on FAO and OXPHOS. Overall, our results indicate that VEN and 8-Cl-Ado can cooperate in targeting rRNA synthesis and OXPHOS and in decreasing the survival of the LSC-enriched cell population, suggesting the VEN/8-Cl-Ado regimen as a promising therapeutic approach for patients with R/R AML.

## 1. Introduction

Chemotherapy induction therapy followed by consolidation therapy with allogeneic stem cell transplantation (alloSCT) is a treatment strategy with the highest chance for long-term survival for the majority of patients with acute myeloid leukemia (AML). However, older and/or unfit patients and those with other medical comorbidities are frequently not candidates for this treatment program. In addition, disease relapse still occurs in a relatively large percentage of patients undergoing alloSCT. Recently, several molecularly targeted therapies have been approved by the US Food and Drug Administration (FDA) for the treatment of AML [1,2,3,4]. Among them is venetoclax (VEN), a selective BCL-2 inhibitor with modest clinical efficacy as a single agent in AML patients, but with a relatively high response rate when combined with hypomethylating agents (HMA; azacitidine or decitabine) or low-dose cytarabine [5,6]. VEN in combination with these agents has demonstrated initial response rates of approximately 60–70% in older and/or unfit newly diagnosed AML patients [5,7,8], and approximately 50% in those with refractory/relapsed (R/R) disease [8,9,10]. Despite these encouraging clinical results, many AML patients that receive VEN treatment in combination either do not respond [5] or eventually relapse. Thus, more effective and less toxic treatment options are urgently needed.

Treatment refractoriness or disease relapse of AML patients is broadly attributed to the persistence of the treatment-resistant quiescent leukemia stem cells (LSCs) [11]. Recently, it has been shown that LSCs differ from normal hematopoietic stem cells (HSCs) in the process of energy production, thus offering a potential strategy to specifically target LSCs [2,12,13]. In fact, while normal HSCs utilize both glycolysis and oxidative phosphorylation (OXPHOS) for energy production, LSCs are highly dependent on OXPHOS that is severely impaired by BCL-2 inhibition through VEN [13]. Of note, metabolic differences have also been reported between LSCs from de novo and those from R/R AML patients, with the former preferentially utilizing BCL-2-dependent amino acid-driven OXPHOS and the latter also utilizing FAO-driven OXPHOS [2,14].

Nucleoside analogs have long represented a backbone in the treatment of AML. Emerging novel nucleoside analogs possess novel mechanism(s) of action and metabolic properties. Furthermore, 8-chloro-adenosine (8-Cl-Ado) is one such analog that is RNA-directed and targets LSCs without significantly inhibiting HSCs [15,16], inhibits FLT3-ITD signaling and has shown anti-neoplastic activity in vitro and in vivo, including in AML [15,16,17,18]. The unique mechanisms of action of 8-Cl-Ado, as well as data from a phase I clinical trial of 8-Cl-Ado monotherapy in R/R AML, prompted us to investigate the combination of VEN plus 8-Cl-Ado in AML.

We previously reported that 8-Cl-Ado synergizes with VEN in the inhibition of energy metabolism and survival of LSC-enriched blast cells [15,19]. Herein, we show that 8-Cl-Ado downregulates the transcription initiation factor TIF-IA, followed by a decrease in ribosomal RNA (rRNA) synthesis and concurrent upregulation of the pro-apoptotic protein p53. An unexpected consequence of exposure to 8-Cl-Ado is p53-induced FAO that self-limits the activity of this drug on LSCs. To this end, VEN has been reported to inhibit FAO and OXPHOS in LSC-enriched AML cells and therefore may overcome the otherwise p53-dependent self-limiting effect of 8-Cl-Ado. Herein, we demonstrate that by regulation of both rRNA synthesis and FAO/OXPHOS metabolism, the combination of VEN and 8-Cl-Ado synergistically inhibits LSC-enriched AML cells, suggesting VEN plus 8-Cl-Ado as a potential novel treatment regimen for AML.

## 2. Materials and Methods

Human samples. Human specimens were collected from patients registered at City of Hope (COH) National Medical Center who had consented to the City of Hope Institutional Review Board approved protocol (IRB#18067); specimens from healthy donors were collected under COH IRB#06229. The study was conducted in accordance with the Declaration of Helsinki. Patient characteristics of primary AML samples are listed in Supplementary Table S1.

RT-PCR and q-PCR analysis. To measure pre-rRNA and GAPDH expression, total RNA was extracted using the RNeasy Mini Kit (Qiagen, Valencia, CA, USA). For pre-rRNA and GAPDH expression, first-strand cDNA was synthesized using the SuperScript III First-Strand Kit. q-PCR was performed using TaqMan Gene Expression Assays (Thermo Fisher, Waltham, MA, USA). GAPDH was used as an internal control and the results are presented as log2-transformed ratio according to the 2^–ΔCt^ method (ΔCt = Ct of target -Ct of reference; Ct = cycle threshold). Primer sequences for q-PCR are listed in Supplementary Table S3.

ChIP assay. Chromatin immunoprecipitation (ChIP) was performed as described by the manufacturer (Pierce, Appleton, WI, USA). Precleared chromatin was incubated overnight by rotation with 4 μg of Pol I antibody or IgG antibody as a negative control. Immunoprecipitates were resuspended in 50 μL TE buffer. Inputs and immunoprecipitated DNA samples were quantified by q-PCR on a 7900T Fast real-time PCR system (Applied Biosystems, Waltham, MA, USA). Antibodies are listed in Supplementary Table S4. Primers are listed in Supplementary Table S3.

RNA labeling and analysis. The cells were washed and incubated in phosphate-free DMEM (Gibco) supplemented with 10% FBS for 2 h, followed by one-hour labeling with 0.5 mCi [^32^P] orthophosphate (PerkinElmer, Waltham, MA, USA). Total RNA was extracted with TRIzol (Life Technology, Carlsbad, CA, USA) according to the manufacturer’s protocol. Equal amounts of RNA (10 μg) were separated on a 1.2% MOPS formaldehyde gel. The gel was dried and visualized by autoradiography.

Immunocytochemistry-FISH. For immunofluorescence–fluorescence in situ hybridization (IF-FISH), the cells were washed in 1x DPBS and fixed in 3.7% paraformaldehyde, 0.2% Triton X-100 and 1x DPBS for 10 min and incubated for 1 h at 37 °C in 10% blocking solution. After washing, the cells were incubated with primary antibodies (anti-TIF-IA and anti-UBF) for 1 h and secondary anti-mouse/rabbit-Alexa 594/488 goat antibodies (Thermo Fisher) for an additional 1 h, at 37 °C. The cells were denatured at 80 °C for 6 min and incubated overnight with probe-hybridizing buffer. Nuclei were counterstained with 4,6-diamidino-2-phenylindole (DAPI). Cell images were acquired using a Zeiss LSM880 confocal microscope (Carl Zeiss, Jena, Germany). Antibodies are listed in Supplementary Table S4. 

Assessment of apoptosis using flow cytometry. The Annexin-V and DAPI double staining method was used to evaluate apoptosis by flow cytometry. Cells were harvested and washed twice with Annexin-V binding buffer (BD Bioscience, San Jose, CA, USA) and resuspended in 100 μL of the same buffer containing Annexin-V APC (BD Bioscience, San Jose, CA, USA). Cells were then incubated in the dark at room temperature for 15 min, washed again and resuspended in 300 μL of buffer. DAPI (Sigma-Aldrich, St. Louis, MO, USA) was added immediately prior to analysis with a LSR II flow cytometer (BD Bioscience, San Jose, CA, USA). Antibodies are listed in Supplementary Table S4. 

RNA and DNA incorporation assay. AML cell lines were incubated with 0–10 μM 8-Cl-Ado for 24 h. Mononuclear cells derived from peripheral blood of AML patients were incubated with 10 μM 8-Cl-Ado for 24 h. For RNA synthesis, cell cultures were incubated with 1 μCi [^3^H]-uridine (Moravek Biochemicals, Brea, CA, USA) per 1 mL for 1 h. For DNA synthesis, cell cultures were incubated with 5 μCi [^3^H]-thymidine (Moravek Biochemicals) per 2 mL for 3 h. The cell cultures were then transferred onto Whatman GF/C glass microfiber filters (GE Healthcare) pre-treated with 1% aqueous sodium pyrophosphate on a multiscreen vacuum assay system (Millipore Corp., Bedford, MA, USA). The filters were then washed with cold PBS, followed by two washes with 0.4 N perchloric acid, then dried with 70% ethanol. The glass filters were then added to a scintillation vial with 7 mL scintillation fluid; the level of radioactivity was measured using a liquid scintillation analyzer (Packard Instrument Co., Downers Grove, IL, USA) and expressed as percent of control.

Gene set enrichment analysis. RNA-seq, RNA expression analysis and gene set enrichment analysis (GSEA). For high-resolution genomic profiling (mRNA-seq) to evaluate mRNA profiles, sequencing was performed on an Illumina Hiseq 2500. Reads were trimmed to remove poly(A) tail and Illumina adapter using Trimmomatics, then aligned to human genome hg38 using Star v2.6.0 with default settings. Expression levels of ensemble genes were counted using HTseq-count. Custom R scripts and Bioconductor packages “edgeR” were used for data normalization and inter-group comparisons. GSEA v4.0 was used to identify Gene Ontology (GO) and canonical pathways (downloaded from Msigdb v7.0) that are altered upon drug treatment vs. control or vs. VEN.

Statistical analysis. To compare the means of 2 groups, results were generally compared by using unpaired, two-tailed Student’s *t*-test, with values from at least 2 independent experiments with triplicate determination, unless otherwise stated. Data are presented as mean ± standard error (S.E.), as indicated. *p* < 0.05 was considered statistically significant; ns = not significant. All statistical analyses were conducted using SigmaPlot 12.5 (Systat Software, Chicago, IL, USA). All statistical tests were two-sided.

## 3. Results

### 3.1. 8-Cl-Ado Inhibits RNA Synthesis Rather Than DNA Synthesis in AML Cell Lines and Primary Blasts

**Figure 1:**
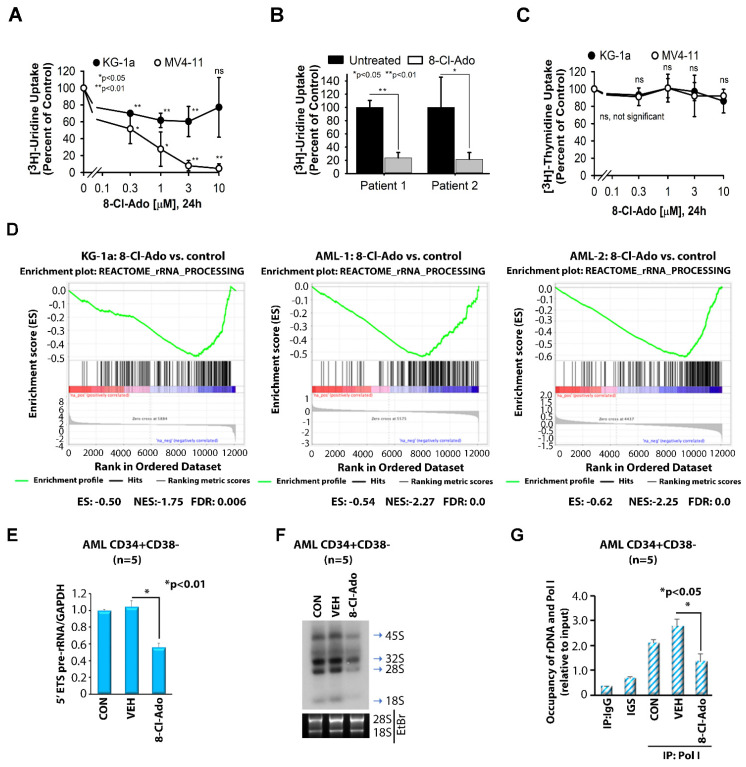
Regulation of rRNA synthesis by 8-Cl-Ado. (**A**,**B**) Effect of 8-Cl-Ado on RNA synthesis in AML cells lines (**A**) and primary AML blast cells (**B**). Cell lines KG-1a and MV4-11 were incubated with 0, 0.3, 1, 3 or 10 μM 8-Cl-Ado for 24 h. Primary blast cells from 2 patients were incubated with or without 10 μM 8-Cl-Ado for 24 h. In the last hour, [^3^H]-uridine was added to measure uptake into newly synthesized RNA. (**C**) Effect of 8-Cl-Ado on DNA synthesis in AML cell lines KG-1a and MV4-11. Cells were incubated with 0, 0.3, 1, 3 or 10 μM 8-Cl-Ado for 24 h. In the last 3 h, [^3^H]-thymidine was added to measure uptake of newly synthesized DNA. *n* = 1 (in triplicate). Error bars = standard error. (**A**–**C**) *n* = 1 (in triplicate, KG-1a; in duplicate, MV4-11 and primary AML). (**D**) Gene set enrichment analysis (GSEA) graphs of genes involved in rRNA synthesis upon treatment of KG-1a and two primary AML cells with 500 nM 8-Cl-Ado for 24 h. *n* = 2. ES enrichment score, NES normalized enrichment score, FDR false discovery rate. (**E**–**G**) LSC-enriched blasts (*n* = 5) were treated with vehicle control (VEH) or 500 nM 8-Cl-Ado for 24 h. RNA was extracted for measurement of 5′ ETS pre-rRNA (**E**) or for radioactive labeling with [^32^P] (**F**). ChIP assay was performed to measure the level of Pol I recruited to the rDNA promoter. Original blots see Supplementary File S1 (**G**). GAPDH (**E**) or 10% input (**G**) was used as the internal control. (**E**,**G**) *n* = 2, in triplicate. CON, control; VEH, vehicle; 8-Cl-Ado, 8-chloro-adenosine.

In contrast to traditional nucleoside analogs that contain deoxyribose or arabinose sugars, 8-Cl-Ado has a ribose sugar and predominantly incorporates into newly transcribed RNA rather than DNA, causing RNA chain termination and cell death [17,20]. To evaluate the effects of 8-Cl-Ado on RNA synthesis in AML, KG-1a and MV4-11 cells as well as AML patients’ primary blasts were incubated in vitro with 8-Cl-Ado for 24 h and pulsed with [^3^H]-labeled uridine, prior to evaluation via scintillation counting. As compared to vehicle-treated controls, RNA synthesis decreased in a dose-dependent manner in both KG-1a and MV4-11 cell lines (Figure 1A) as well as in primary AML blasts (Figure 1B). In both cell lines, inhibition of RNA synthesis was detected at concentrations as low as 300 nM 8-Cl-Ado. The response was more pronounced in FLT3-ITD-positive MV4-11 cells, where RNA synthesis was inhibited to ~50% relative to control after 24 h exposure to 300 nM 8-Cl-Ado and was further reduced to ~25% at 1 μM 8-Cl-Ado. In primary AML blasts exposed to 8-Cl-Ado at a concentration as high as 10 µM, >70% inhibition of RNA synthesis was observed. In contrast, significant changes in DNA synthesis were not observed in either cell line with exposure to concentrations as high as 10 μM 8-Cl-Ado as assessed by the incorporation of radioactive thymidine (Figure 1C). These results suggest that 8-Cl-Ado is a nucleoside analog targeting RNA synthesis in leukemia cells [16,17,20]. For 8-Cl-Ado in AML, we have previously reported toxicity studies, effects on malignant and normal hematopoietic stem cells and effects on tumor growth in xenografted animals [15]. Effects of 8-Cl-Ado on other cancers, including multiple myeloma, breast cancer and colon cancer, have also been reported [16,17,18,20].

### 3.2. 8-Cl-Ado Inhibits Ribosomal RNA Synthesis in the Leukemic Stem Cell-Enriched Population

Actively growing cells depend on the continuous production of large amounts of ribosomes, including rRNA [21]. RNA polymerase I (Pol I) accounts for up to 60% of the total RNA synthesized in the nucleus and transcribes the precursors of the three largest species of rRNA, 28S, 18S and 5.8S, but not 5S rRNA [22]. Elevated levels of rRNA represent a common feature of cancer cells, and numerous reports from others and us indicate that rRNA synthesis is important for cancer and leukemia cell proliferation [23,24,25,26,27,28]. Since 8-Cl-Ado inhibits RNA synthesis, we postulated that this drug also affected rRNA synthesis. Gene set enrichment analysis (GSEA) of KG-1a AML cells treated with 8-Cl-Ado for 24 h demonstrated that 8-Cl-Ado significantly suppresses a gene set involved in rRNA synthesis (*p*-value, 0.0; FDR, 0.006; Figure 1D, left), as compared to vehicle-treated controls. Treatment of primary AML blasts with 500 nM 8-Cl-Ado for 24 h also significantly induced negative enrichment of the rRNA synthesis genes (Figure 1D, middle and right).

To further explore the effects of 8-Cl-Ado on rRNA synthesis in LSCs, we incubated enriched CD34+CD38− primary AML blasts with 500 nM 8-Cl-Ado for 24 h. We observed significantly suppressed pre-rRNA synthesis as determined by the reduced level of 5′ external transcribed spacer (ETS) pre-rRNA (Figure 1E) and decreased rRNA synthesis demonstrated by [^32^P]-labeling of RNA (Figure 1F). Given that Pol I recruitment to rDNA is essential for rRNA transcription [23,24,26,27,28,29], we also demonstrated that 8-Cl-Ado treatment reduced the recruitment of Pol I to rDNA by chromatin immunoprecipitation (ChIP) assay (Figure 1G). Taken together, these results indicate that 8-Cl-Ado inhibits rRNA synthesis in AML cells.

### 3.3. 8-Cl-Ado Inhibits Ribosomal RNA Synthesis through Downregulation of TIF-IA

**Figure 2:**
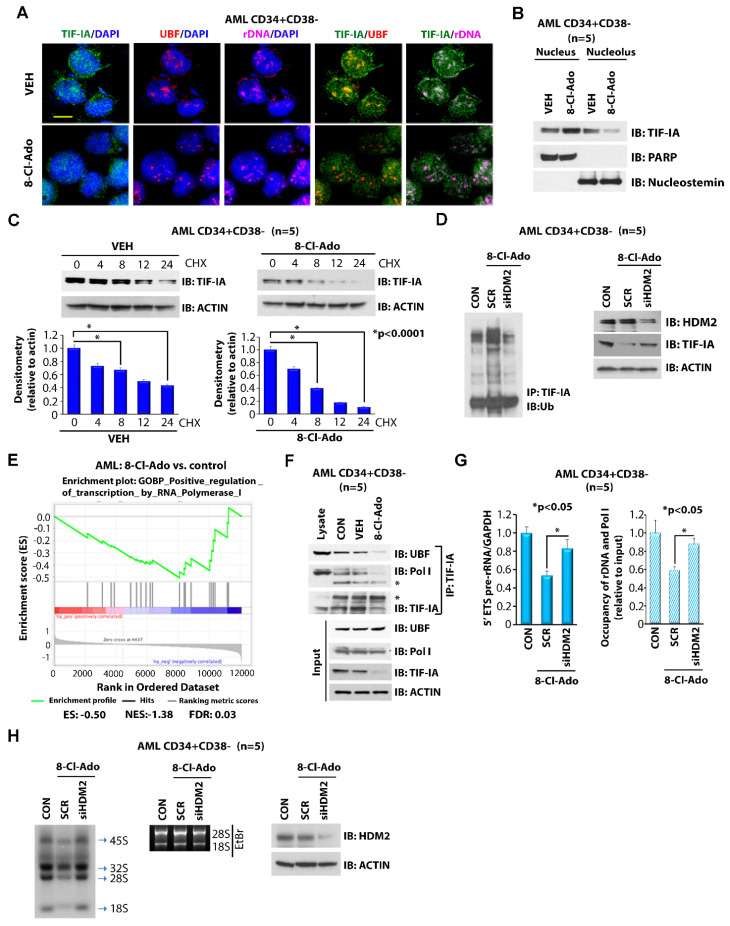
Regulation of TIF-IA expression and function by 8-Cl-Ado. (**A**) Effects of 8-Cl-Ado on cellular distribution of TIF-IA. LSC-enriched blasts were treated with vehicle control (VEH) or 500 nM 8-Cl-Ado for 24 h prior to staining with anti-TIF-IA, anti-UBF antibodies and rDNA probe by immunofluorescence-FISH assay. Images are visualized for TIF-IA, UBF and rDNA using confocal immunofluorescence microscopy. Scale bar, 10 µm. (**B**) The cells were treated as described in (**A**) and fractionated into nucleus and nucleolus fragments and each lysate was immunoblotted with the indicated antibodies. (**C**) Effect of 8-Cl-Ado on TIF-IA stability. LSC-enriched blasts were treated with vehicle control (VEH) or 500 nM 8-Cl-Ado for 24 h. The cells were then treated with 10 μM of cycloheximide (CHX) for the indicated times and each lysate was immunoblotted with anti-TIF-IA and anti-actin antibodies. (**D**) Effect of HDM2 knockdown on 8-Cl-Ado-induced TIF-IA ubiquitination. LSC-enriched blasts were treated with 500 nM 8-Cl-Ado for 24 h in the presence of siSCR control or siHDM2. Left, lysate was immunoprecipitated using anti-TIF-IA and immunoblotted with anti-ubiquitin (Ub) antibodies. Right, immunoblotting control with anti-HDM2, anti-TIF-IA and anti-actin antibodies. (**E**) Gene set enrichment analysis (GSEA) graph of genes involved in Pol I-regulated transcription upon treatment of primary AML cells with 500 nM 8-Cl-Ado for 24 h. *n* = 2. ES enrichment score, NES normalized enrichment score, FDR false discovery rate. (**F**) Effect of 8-Cl-Ado on TIF-IA interaction with UBF and Pol I. LSC-enriched blasts were treated with vehicle control (VEH) or 500 nM 8-Cl-Ado for 24 h. Lysate was immunoprecipitated using anti-TIF-IA and immunoblotted with anti-UBF and anti-Pol I antibodies. (**G**,**H**) Effects of HDM2 KD on 8-Cl-Ado-regulated rRNA synthesis. LSC-enriched blasts were transfected with siSCR or siHDM2 (40 nM) for 12 h and treated with 8-Cl-Ado for additional 24 h. (**G**) Left, RNA was extracted for measurement of 5′ ETS pre-rRNA. Right, ChIP assay using anti-Pol I antibody and primers for rDNA. *n* = 2, in triplicate. (**H**) Left side, RNA was extracted to measure [^32^P] in an RNA labeling assay with. Right side, HDM2 KD efficacy is shown. CON, control; VEH, vehicle; SCR, scramble siRNA; 8-Cl-Ado, 8-chloro-adenosine. Target sequences for siRNAs are listed in Supplementary Table S2. Original blots see Supplementary File S1.

We next dissected the molecular mechanism through which 8-Cl-Ado deregulated rRNA synthesis in AML cells. Transcription of rDNA by Pol I requires a pre-initiation complex consisting of RNA Pol I, upstream binding factor (UBF), selectivity factor 1 (SL-1) and TIF-IA transcription initiation factor. While TIF-IA is required to recruit Pol I to the rDNA promoter, UBF and SL-1 are primarily responsible for DNA binding [29,30]. We observed that 8-Cl-Ado treatment of LSC-enriched primary AML cells disrupted the colocalization of TIF-IA and UBF at the rDNA site, as demonstrated by immunofluorescent and fluorescent in situ hybridization (IF-FISH) with anti-TIF-IA, anti-UBF antibodies and rDNA probes (Figure 2A). The cellular fractionation assay also demonstrated that 8-Cl-Ado treatment resulted in a decline in TIF-IA in the nucleolar fraction with a parallel increase in the nucleus (Figure 2B). Since TIF-IA is a downstream substrate of HDM2 ubiquitin ligase-induced ubiquitylation and degradation [27], we also examined the effects of 8-Cl-Ado on TIF-IA ubiquitylation and stability. Here, 8-Cl-Ado induced the ubiquitylation (Figure 2D) and degradation of TIF-IA (Figure 2C), while knocking down (KD) HDM2 mRNA using siRNA partially rescued the effects (Figure 2D). Of note, 8-Cl-Ado treatment of primary AML cells resulted in the significant inhibition of a gene set involved in transcription regulation by RNA Pol I (FDR 0.03) (Figure 2E), suggesting that 8-Cl-Ado inhibits proper formation of the Pol I pre-initiation transcription complex through downregulation of TIF-IA. To this end, 8-Cl-Ado-induced inhibition of TIF-IA disrupted the rRNA transcription pre-initiation complex, as demonstrated by a decreased interaction of Pol I, UBF and TIF-IA (Figure 2F), leading to the inhibition of Pol I/rDNA binding and rRNA synthesis (Figure 2G,H). Pre-treatment with siRNA against HDM2 (siHDM2) prevented the 8-Cl-Ado-induced downregulation of TIF-IA (Figure 2D), thereby rescuing Pol I/rDNA binding and rRNA synthesis (Figure 2G,H). Taken together, our results indicate that the inhibitory effects of 8-Cl-Ado on rRNA synthesis are mediated through the down-regulation of the TIF-IA protein.

### 3.4. Inhibition of TIF-IA by 8-Cl-Ado Induces p53 Apoptotic Signaling and Concurrently Activates p53-Regulated FAO in the Leukemic Stem Cell-Enriched Population

**Figure 3:**
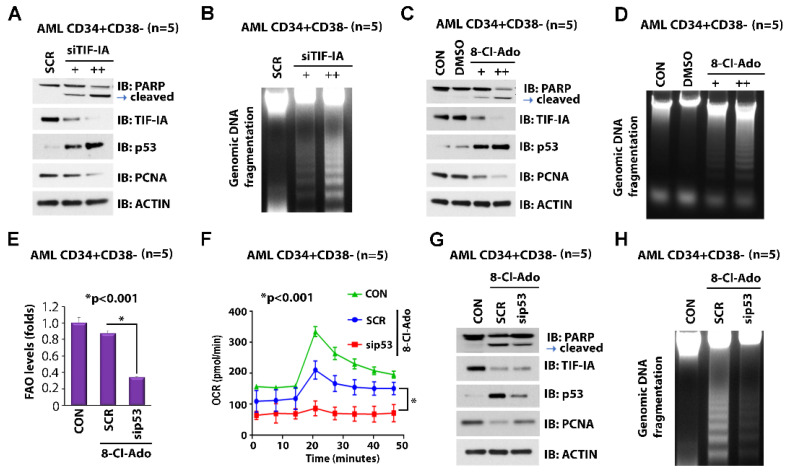
Effects of 8-Cl-Ado on p53 expression and p53-regulated OXPHOS metabolism. (**A**,**B**) Effects of TIF-IA KD on expression of p53 and PCNA and on apoptosis. LSC-enriched blasts were transfected with siSCR or siTIF-IA [20 nM (+) and 40 nM (++)] for 24 h. (**A**) Each lysate was immunoblotted with the indicated antibodies. (**B**) Genomic DNA was isolated for DNA fragmentation assay. (**C**,**D**) Effects of 8-Cl-Ado on levels of TIF-IA, p53, PCNA protein and apoptosis. LSC-enriched blasts were treated with DMSO control or 8-Cl-Ado [500 nM (+) and 1 µM (++)] for 24 h. (**C**) Each lysate was immunoblotted with the indicated antibodies. (**D**) DNA fragmentation assay. (**E**,**F**) Effects of 8-Cl-Ado on fatty acid oxidation (FAO) and OCR (OXPHOS). LSC-enriched blasts were transfected with siSCR or sip53 (20 nM) for 12 h. The cells were then treated with 500 nM 8-Cl-Ado for additional 24 h. (**E**) Levels of FAO were measured by the oxidation rate of ^3^H-palmitic acid. *n* = 2, in triplicate. (**F**) Levels of oxidative consumption rate (OCR) were measured by seahorse cell energy testing assay. *n* = 2, in triplicate. (**G**,**H**) Effects of p53 KD on 8-Cl-Ado-regulated apoptosis. LSC-enriched blasts were transfected with siSCR or sip53 (20 nM) for 12 h. The cells were then treated with 500 nM 8-Cl-Ado for additional 24 h. (**G**) Each lysate was immunoblotted with the indicated antibodies. (**H**) DNA fragmentation assay. CON, control; SCR, scramble siRNA; 8-Cl-Ado, 8-chloro-adenosine. Target sequences for siRNAs are listed in Supplementary Table S2. Original blots see Supplementary File S1.

Our previous work indicated that TIF-IA negatively regulates p53 expression [29]. In LSC-enriched AML blasts, depletion of TIF-IA with siRNA resulted in increased p53 levels and apoptosis, as shown by PARP cleavage (Figure 3A) and genomic DNA (gDNA) fragmentation (Figure 3B). Consistent with increased levels of apoptosis, proliferating cell nuclear antigen (PCNA) protein levels were reduced (Figure 3A). In addition to decreased TIF-IA and PCNA protein levels, we observed increased p53 levels and apoptosis in a dose-dependent manner in 8-Cl-Ado-treated LSC-enriched blasts (Figure 3C,D).

We previously reported that 8-Cl-Ado treatment of LSCs resulted in decreased FAO and a diminished oxygen consumption rate (OCR), a marker for OXPHOS, but did not significantly alter the extracellular acidification rate (ECAR), a marker for glycolysis [19]. Of note, compared to the regular effective dose of 500 nM in AML cell lines and primary blasts, a higher concentration of 1 µM single-agent 8-Cl-Ado was required for the significant suppression of FAO and OCR and induction of apoptosis in LSC-enriched blasts (Figure 3C,D) [19]. Previous studies reported that p53 promotes OXPHOS [31]. LSCs, in contrast to HSCs, are deficient in glycolysis and dependent on OXPHOS for energy production [32]. As mentioned above, 8-Cl-Ado diminishes FAO and OXPHOS but also induces p53 expression, which in turn can activate FAO. Thus, we postulated that the effect of 8-Cl-Ado-mediated p53 expression in inducing apoptosis may be self-limiting through the enhancement of FAO and OCR/OXPHOS in LSCs. In fact, knockdown of p53 mRNA by siRNA augmented the inhibitory effects of 8-Cl-Ado on FAO and OCR in LSC-enriched blasts (Figure 3E,F) but also reduced apoptosis (Figure 3G,H). These data suggest that the anti-leukemic effects of 8-Cl-Ado via the inhibition of rRNA synthesis and induction of p53-dependent apoptotic signaling may be self-limited through the FAO-activating effect of p53.

### 3.5. VEN Antagonizes p53-Induced Activation of FAO and OXPHOS

**Figure 4:**
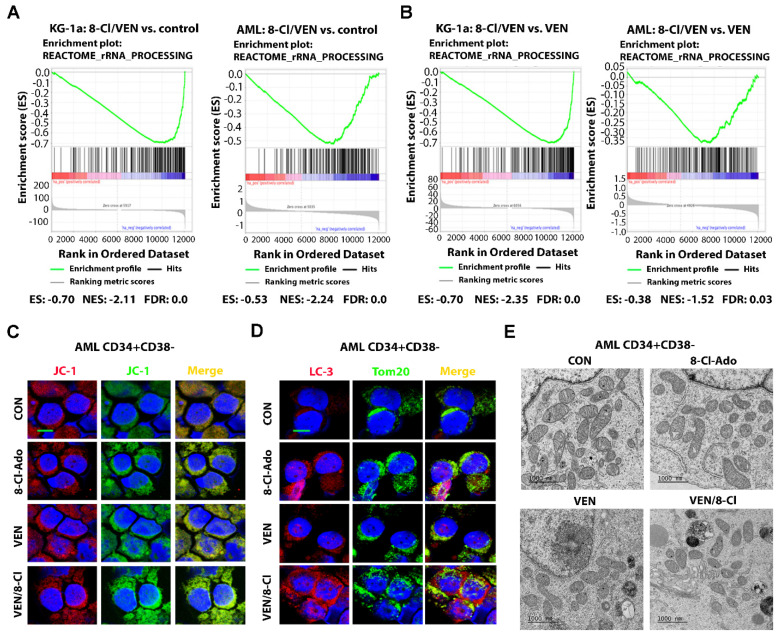
Effects of 8-Cl-Ado plus VEN on rRNA synthesis and mitochondria metabolism. LSC-enriched blasts and KG-1a cells were treated with 500 nM 8-Cl-Ado, 10 nM VEN or both. (**A**,**B**) Gene set enrichment analysis (GSEA) graphs of genes involved in rRNA synthesis upon treatment of KG-1a and primary AML blast cells for 24 h. GSEA of rRNA synthesis for direct comparison of VEN/8-Cl-Ado versus control (**A**) or VEN (**B**). *n* = 2. ES enrichment score, NES normalized enrichment score, FDR false discovery rate. (**C**) Mitochondrial membrane potential was measured by staining of the treated cells with JC-1 probe. The red and green fluorescence is indicative of JC-1 polymer and monomer, respectively. Scale bar, 10 µm. (**D**) Mitophagy marker LC3 and mitochondria marker TOM20 were measured by immunofluorescence staining. Scale bar, 10 µm. (**E**) Mitochondria morphology was measured by electron microscope imaging. Scale bar, 1000 nm. CON, control; VEH, vehicle; VEN, venetoclax; 8-Cl-Ado, 8-chloro-adenosine.

**Figure 5:**
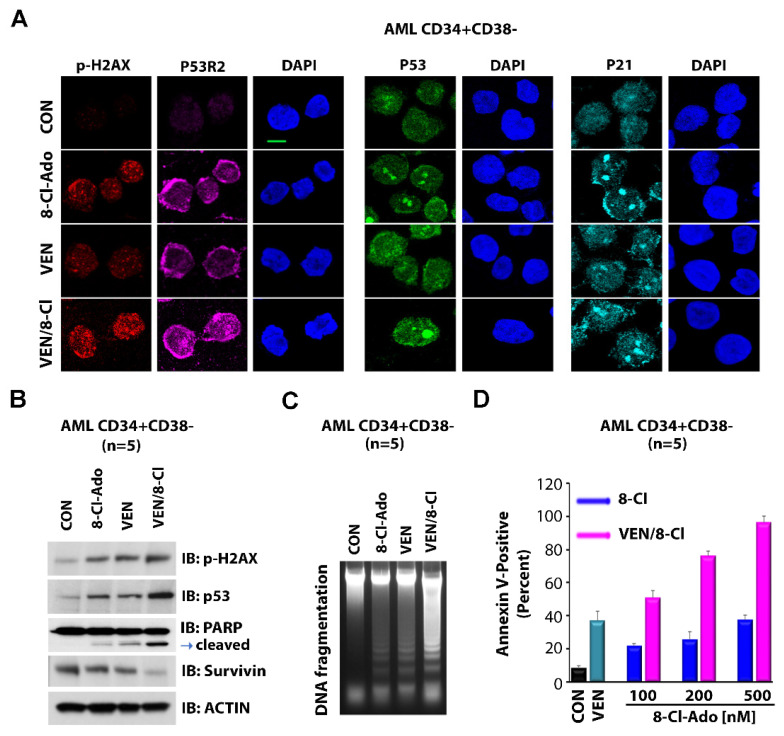
Effects of 8-Cl-Ado plus VEN on p53 signaling and apoptosis. LSC-enriched blasts were treated with 500 nM 8-Cl-Ado, 10 nM VEN or both. (**A**,**B**) Levels of p-H2AX, p53 and p21 proteins were measured by immunofluorescence and immunoblotting. Original blots see Supplementary File S1. (**C**,**D**) Levels of apoptosis as determined by DNA fragmentation (**C**) and flow cytometry for Annexin V (**D**). (**D**) *n* = 2, in triplicate. CON, control; VEN, venetoclax; 8-Cl-Ado, 8-chloro-adenosine.

We recently reported that VEN and 8-Cl-Ado cooperate in the inhibition of OXPHOS and induction of apoptosis in LSC-enriched blasts [19]. The 8-Cl-Ado/VEN combination synergistically inhibited the growth and survival of AML cells in vitro and increased the survival of immune-deficient, AML-bearing mice [19]. Treatment with single-agent 8-Cl-Ado alone resulted in an increase in the expression of apoptotic protein p53. Importantly, p53 expression was associated with the p53-induced activation of FAO and OXPHOS, thus diminishing the inhibitory effect of 8-Cl-Ado on FAO and OXPHOS. Thus, we postulated that the addition of VEN to 8-Cl-Ado may augment the anti-metabolic effect of 8-Cl-Ado on LSCs by inhibiting levels of FAO and OXPHOS. GSEA results obtained after treatment of KG-1a and primary AML cells with VEN and 8-Cl-Ado demonstrated that the combined treatment significantly downregulates a gene set associated with rRNA synthesis, as compared to non-treated control (Figure 4A) or VEN-only (Figure 4B) treatment. As demonstrated by FAO and OCR/OXPHOS assays, the addition of VEN to 8-Cl-Ado treatment further suppressed FAO and OXPHOS, as compared to single-agent treatment (Supplementary Figure S1A,B) [19]. Using immunofluorescence staining and electron microscopy, we also observed that combined treatment with 8-Cl-Ado and VEN further decreased mitochondrial metabolism in LSC-enriched blasts. Mitochondrial membrane potential was measured by immunostaining with the fluorescent mito-probe JC-1, where the ratio of red (aggregates of JC-1) to green (JC-1 monomers) fluorescence intensity indicates “healthy” vs. “unhealthy” mitochondria. As shown in Figure 4, the VEN/8-Cl-Ado combination further decreased mitochondrial membrane potential, as shown by the ratio of red JC-1 (polymer) to green JC-1 (monomer) fluorescence (Figure 4C and Figure S2) [33] and increased expression of mitophagy marker LC3 (Figure 4D), a ubiquitin-like protein involved in protein degradation. Compared to the control, treatment with single-agent 8-Cl-Ado or VEN increased mitochondrial fragmentation, a process associated with cell death (Figure 4E) [34]. Combination treatment with VEN/8-Cl-Ado further increased mitochondria fragmentation, as shown by the reduction in mitochondria size (Figure 4E). These results suggested that the addition of VEN to 8-Cl-Ado counteracts the FAO-activating effect of p53, thus inhibiting mitochondrial metabolism and the survival of LSCs. Of note, the VEN/8-Cl-Ado combination also deregulated mitochondria’s morphology and function. Accordingly, our results demonstrate that the VEN/8-Cl-Ado combination induced p53 apoptotic signaling, as supported by the induction of p53 and p53-inducible ribonucleotide reductase subunit p53R2 and activation of the p53 downstream target p21 (Figure 5A). Although the VEN/8-Cl-Ado combination did not induce a significant increase in the nucleolar translocation of p53 and p21, as compared to 8-Cl-Ado single agent (Figure 5A), we observed significant p53 pathway activation and apoptosis, as shown by Western blot, DNA fragmentation and Annexin V staining, compared to single agents (Figure 5B–D). The combination treatment induced DNA damage marker p-H2AX and PARP cleavage and inhibited Survivin expression (Figure 5B), increased DNA fragmentation (Figure 5C) and dose-dependently increased apoptosis (Figure 5D).

### 3.6. 8-Cl-Ado and VEN Induce Apoptosis in Relapsed AML by Targeting rRNA Synthesis and FAO/OXPHOS

**Figure 6:**
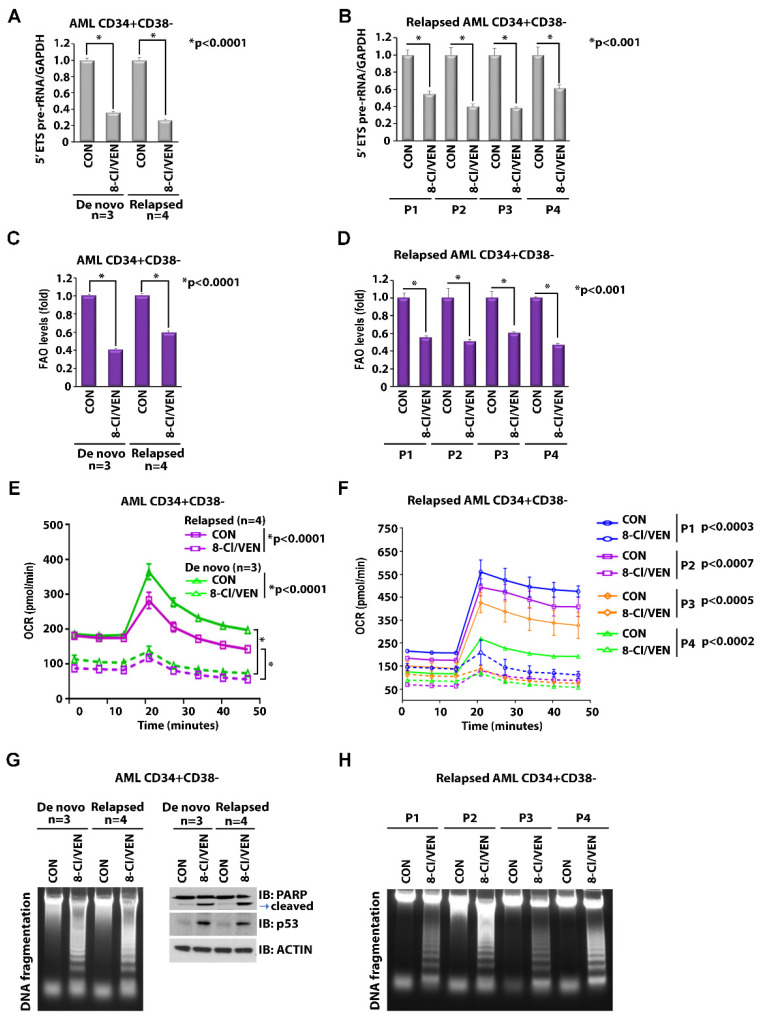
Effects of 8-Cl-Ado plus VEN on rRNA synthesis, OXPHOS metabolism and apoptosis in de novo and relapsed LSCs. LSC-enriched blasts isolated from de novo (*n* = 3) and relapsed (*n* = 4) primary AML cells were treated for 24 h with 10 nM VEN plus 500 nM 8-Cl-Ado. (**A**,**B**) Levels of rRNA synthesis as measured by 5′ETS pre-rRNA. The levels of rRNA synthesis of pooled samples are shown in (**A**) and individual samples of relapsed AML are shown in (**B**). (**C**,**D**) Levels of FAO. The levels of FAO of pooled samples are shown in (**C**) and individual samples of relapsed AML are shown in (**D**). (**E**,**F**) Levels of OCR. The levels of OCR of pooled samples are shown in (**E**) and individual samples of relapsed AML are shown in (**F**). (**A**–**F**) *n* = 2, in triplicate. (**G**,**H**) Levels of apoptosis. The levels of apoptosis as demonstrated by DNA fragmentation, PARP cleavage and p53 expression of pooled samples are shown in (**G**) and individual samples of relapsed AML are shown in (**H**). CON, control; VEN, venetoclax; 8-Cl-Ado, 8-chloro-adenosine. Original blots see Supplementary File S1.

Previous studies showed that LSCs from de novo AML patients are susceptible to the VEN/azacitidine combination, whereas LSCs derived from patients with R/R AML are less sensitive to this treatment [2,10,35]. Of note, it has previously been demonstrated that LSCs from patients with R/R AML, but not with de novo AML, can utilize FAO as an additional metabolic pathway to fuel OXPHOS [11]. Thus, LSCs from R/R AML patients may have a rescuing pathway to antagonize the effects of the VEN-induced loss of BCL-2-dependent, amino acid-driven OXPHOS. We recently demonstrated that the VEN/8-Cl-Ado combination targets FAO and OXPHOS in primary blasts from patients with R/R AML [19]. To investigate the effects of the VEN/8-Cl-Ado combination treatment on LSC-enriched blasts from de novo and relapsed AML patients, we treated CD34+CD38− AML blasts from three patients with de novo AML and four patients with R/R AML with 10 nM VEN and 500 nM 8-Cl-Ado, for 24 h. The VEN/8-Cl-Ado combination treatment suppressed levels of rRNA synthesis (Figure 6A,B), FAO (Figure 6C,D) and OXPHOS (Figure 6E,F) in de novo and relapsed LSC-enriched blast cells. In addition, we observed that the VEN/8-Cl-Ado combination treatment induced apoptosis in LSC-enriched blasts from both de novo and R/R AML patients, as demonstrated by DNA fragmentation, induction of p53 and cleavage of PARP (Figure 6G,H). These results suggest that the VEN/8-Cl-Ado combination is effective not only against de novo AML but also against R/R AML.

## 4. Discussion

**Figure 7:**
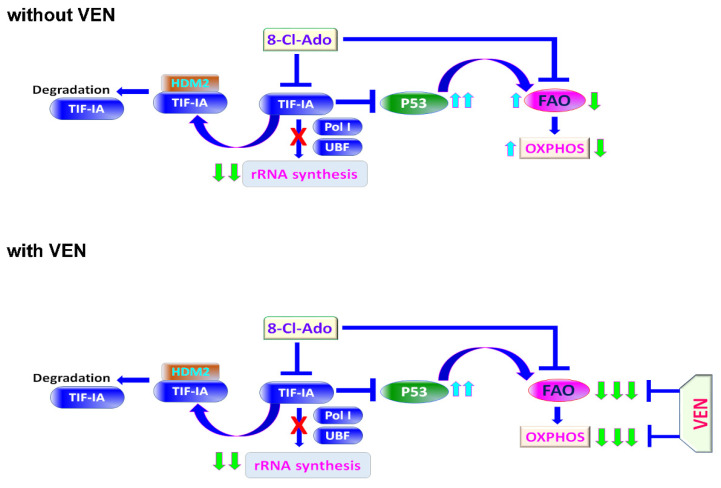
Schematic diagram of a proposed mechanism of synergy of combining 8-Cl-Ado with VEN for targeting of metabolic vulnerabilities in LSC-enriched blasts. Top, in the absence of VEN. Bottom, in the presence of VEN. TIF-IA, transcription initiation factor IA; Pol I, RNA polymerase I; UBF, upstream binding factor; HDM2, human homolog of mouse double minute 2; FAO, fatty acid oxidation; OXPHOS, oxidative phosphorylation; VEN, venetoclax; 8-Cl-Ado, 8-chloro-adenosine.

The schematic diagram (Figure 7) represents a proposed signaling pathway of combining 8-Cl-Ado with VEN for the targeting of metabolic vulnerabilities in LSCs. We hypothesize that 8-Cl-Ado decreases rRNA synthesis, at least in part, through the downregulation and degradation of TIF-IA, while simultaneously increasing the expression of TIF-IA-suppressed p53 protein. While the upregulation of p53 protein expression contributes to cell death, increased p53 also increases FAO and thus OXPHOS and energy production in LSCs. We and others have previously reported that VEN blocks OXPHOS and induces cell death in LSCs [2,19,36,37]. Our new data support the hypothesis that the addition of VEN to 8-Cl-Ado-treated LSCs can overcome the undesirable effect of 8-Cl-Ado on the p53-induced increase in FAO and OXPHOS. Thus, our model demonstrates a mechanism of augmented suppression of LSC survival through the cooperation of VEN and 8-Cl-Ado. The VEN/8-Cl-Ado combination represents a promising novel regimen for the treatment of R/R AML.

VEN combination treatments with either an HMA drug or low-dose cytarabine demonstrate superior clinical outcomes compared to single-agent treatment in AML patients. Relapse of AML is attributed to the presence of the LSC population. Recently, progress has been made in understanding the mechanisms of action of current VEN combination treatments. We and others demonstrated that the VEN/HMA combination augments oxidant stress in AML cells and we provided a molecular mechanism of a VEN-HMA-regulated NF-E2-related factor 2 (Nrf2) antioxidant pathway that could explain the impressive results observed in early clinical studies in AML [38], but mechanisms of resistance remain to be fully elucidated.

Previous studies showed that the VEN/azacitidine combination targets amino acid metabolism/OXPHOS and inhibits LSCs from de novo AML patients; however, LSCs from R/R AML patients can also utilize fatty acid metabolism to feed into OXPHOS and are thus less sensitive to VEN/azacitidine. Importantly, it has been demonstrated that dual inhibition of both amino acid metabolism and fatty acid metabolism using VEN/azacitidine together with the fatty acid transport inhibitor sulfo-N-succinimidyl oleate re-sensitizes resistant AML blasts to VEN/azacitidine treatment [36]. Therefore, the development of new VEN combinations that simultaneously target both amino acid metabolism and fatty acid metabolism represents a therapeutic strategy to durably eradicate LSCs in patients with R/R AML.

We have previously reported that the nucleoside analog 8-Cl-Ado possesses in vitro and in vivo activity against AML as a single agent and that it synergizes with the FLT3-ITD inhibitor quizartinib [15]. Intracellularly, 8-Cl-Ado is converted into its cytotoxic metabolite 8-Cl-ATP by adenosine kinase and mono- and diphospho- kinases. Then, 8-Cl-ATP incorporates into newly synthesized RNA, thereby inhibiting DNA-/RNA-polymerases and causing chain termination and apoptosis [20]. Moreover, 8-Cl-Ado not only targets LSCs but quickly diminishes the bulk of the circulating AML blast cells (our unpublished results from a phase I clinical trial with 8-Cl-Ado in patients with R/R AML). We recently reported that the VEN/8-Cl-Ado combination synergistically targets OXPHOS and inhibits the growth of AML cells in vitro and in vivo [19]. In the current study, we show that the addition of VEN to 8-Cl-Ado not only augments the inhibition of OXPHOS, but also can inhibit FAO, thus providing a rationale for the use of this combination to target otherwise resistant LSCs. Importantly, we have already shown in vivo the strong anti-leukemic activity of 8-Cl-Ado in combination with VEN in an orthotopic mouse model of AML, using the fast-growing FLT3-ITD-positive AML cell line MV4-11 [19], but the mechanistic basis for this synergism remains to be fully elucidated.

rRNA synthesis is a fundamental process utilized by all cells and targeting of this process for therapeutic intervention in cancer was long deemed not feasible. However, the dependence on increased rDNA transcription appears to selectively render cancer cells vulnerable to therapeutic intervention in the rRNA synthesis process [29,39]. In cancer cells, a number of chemotherapeutic drugs have been investigated for their direct or indirect inhibitory effects on rRNA synthesis and its upstream modulators [29,40]. Because rRNA synthesis is upregulated in AML [27,28], suppression of rRNA synthesis by 8-Cl-Ado through the downregulation of TIF-IA expression and Pol I complex formation should have strong inhibitory effects on AML blasts and LSCs. Moreover, the 8-Cl-Ado-mediated downregulation of TIF-IA was accompanied by the activation of p53. In this study, we have used pooled primary samples with heterogeneous genotypes, including different p53 status. It would be interesting to reveal whether the p53 mutation status impacts the effect of the VEN/8-Cl-Ado combination on AML cells. It is also interesting to find out whether the p53 phosphorylation status is involved in the response to VEN/8-Cl-Ado. Further, the expression status of apoptotic factors Puma, Noxa (both p53-dependent), Bcl-2, Bim and Bax should be investigated in future studies. Although p53 is involved in apoptosis induction, in the present study, 8-Cl-Ado-induced p53 upregulation also increased FAO and OCR in LSCs, thus providing evidence of a self-limiting effect of 8-Cl-Ado. The addition of VEN to 8-Cl-Ado was able to overcome the p53-induced activation of FAO and OCR, suggesting that this combination may be active in eliminating the LSC-enriched AML blast subpopulation.

## 5. Conclusions

We demonstrate that the combination of VEN plus 8-Cl-Ado targets LSCs through the inhibition of rRNA synthesis and FAO/OXPHOS metabolic processes, thus potentially also targeting LSCs otherwise resistant to the currently used VEN combinatory regimens. To this end, a phase I/II clinical trial with VEN plus 8-Cl-Ado in patients with R/R AML will be initiated at our institution shortly.

## Data Availability

The bioinformatics raw data/analyzed raw data used in the current study are available from the corresponding authors on reasonable request.

## Supplementary Materials

The following supporting information can be downloaded at: https://www.mdpi.com/article/10.3390/cancers14061446/s1. Supplementary Materials and Methods; Table S1: Patient characteristics of primary AML samples; Table S2: Target sequences for siRNAs; Table S3: Primer sequences used for ChiP and qPCR analysis; Table S4: List of antibodies used for ChiP, IF, IP and IB analysis; Figure S1: Effects of 8-Cl-Ado plus VEN on mitochondria metabolism. Figure S2: Effects of 8-Cl-Ado plus VEN on mitochondria membrane potential. File S1: Western blot raw data and quantification of Western blots.

## References

[1] Bohl S.R., Bullinger L., Rucker F.G. (2019). New Targeted Agents in Acute Myeloid Leukemia: New Hope on the Rise. Int. J. Mol. Sci..

[2] Jones C.L., Stevens B.M., D’Alessandro A., Reisz J.A., Culp-Hill R., Nemkov T., Pei S., Khan N., Adane B., Ye H. (2018). Inhibition of Amino Acid Metabolism Selectively Targets Human Leukemia Stem Cells. Cancer Cell.

[3] Krauss A.C., Gao X., Li L., Manning M.L., Patel P., Fu W., Janoria K.G., Gieser G., Bateman D.A., Przepiorka D. (2019). FDA Approval Summary: (Daunorubicin and Cytarabine) Liposome for Injection for the Treatment of Adults with High-Risk Acute Myeloid Leukemia. Clin. Cancer Res. Off. J. Am. Assoc. Cancer Res..

[4] Norsworthy K.J., Ko C.W., Lee J.E., Liu J., John C.S., Przepiorka D., Farrell A.T., Pazdur R. (2018). FDA Approval Summary: Mylotarg for Treatment of Patients with Relapsed or Refractory CD33-Positive Acute Myeloid Leukemia. Oncologist.

[5] DiNardo C.D., Pratz K., Pullarkat V., Jonas B.A., Arellano M., Becker P.S., Frankfurt O., Konopleva M., Wei A.H., Kantarjian H.M. (2019). Venetoclax combined with decitabine or azacitidine in treatment-naive, elderly patients with acute myeloid leukemia. Blood.

[6] Pollyea D.A., Amaya M., Strati P., Konopleva M.Y. (2019). Venetoclax for AML: Changing the treatment paradigm. Blood Adv..

[7] DiNardo C.D., Pratz K.W., Letai A., Jonas B.A., Wei A.H., Thirman M., Arellano M., Frattini M.G., Kantarjian H., Popovic R. (2018). Safety and preliminary efficacy of venetoclax with decitabine or azacitidine in elderly patients with previously untreated acute myeloid leukaemia: A non-randomised, open-label, phase 1b study. Lancet Oncol..

[8] Wei A.H., Strickland S.A., Hou J.Z., Fiedler W., Lin T.L., Walter R.B., Enjeti A., Tiong I.S., Savona M., Lee S. (2019). Venetoclax Combined With Low-Dose Cytarabine for Previously Untreated Patients With Acute Myeloid Leukemia: Results From a Phase Ib/II Study. J. Clin. Oncol. Off. J. Am. Soc. Clin. Oncol..

[9] Aldoss I., Yang D., Aribi A., Ali H., Sandhu K., Al Malki M.M., Mei M., Salhotra A., Khaled S., Nakamura R. (2018). Efficacy of the combination of venetoclax and hypomethylating agents in relapsed/refractory acute myeloid leukemia. Haematologica.

[10] DiNardo C.D., Rausch C.R., Benton C., Kadia T., Jain N., Pemmaraju N., Daver N., Covert W., Marx K.R., Mace M. (2018). Clinical experience with the BCL2-inhibitor venetoclax in combination therapy for relapsed and refractory acute myeloid leukemia and related myeloid malignancies. Am. J. Hematol..

[11] Felipe Rico J., Hassane D.C., Guzman M.L. (2013). Acute myelogenous leukemia stem cells: From Bench to Bedside. Cancer Lett..

[12] Gilliland D.G., Jordan C.T., Felix C.A. (2004). The molecular basis of leukemia. Hematol. Am. Soc. Hematol. Educ. Program.

[13] Lagadinou E.D., Sach A., Callahan K., Rossi R.M., Neering S.J., Minhajuddin M., Ashton J.M., Pei S., Grose V., O’Dwyer K.M. (2013). BCL-2 inhibition targets oxidative phosphorylation and selectively eradicates quiescent human leukemia stem cells. Cell Stem Cell.

[14] Pollyea D.A., Stevens B.M., Jones C.L., Winters A., Pei S., Minhajuddin M., D’Alessandro A., Culp-Hill R., Riemondy K.A., Gillen A.E. (2018). Venetoclax with azacitidine disrupts energy metabolism and targets leukemia stem cells in patients with acute myeloid leukemia. Nat. Med..

[15] Buettner R., Nguyen L.X.T., Kumar B., Morales C., Liu C., Chen L.S., Pemovska T., Synold T.W., Palmer J., Thompson R. (2019). 8-chloro-adenosine activity in FLT3-ITD acute myeloid leukemia. J. Cell. Physiol..

[16] Stellrecht C.M., Rodriguez C.O., Ayres M., Gandhi V. (2003). RNA-directed actions of 8-chloro-adenosine in multiple myeloma cells. Cancer Res..

[17] Stellrecht C.M., Ayres M., Arya R., Gandhi V. (2010). A unique RNA-directed nucleoside analog is cytotoxic to breast cancer cells and depletes cyclin E levels. Breast Cancer Res. Treat..

[18] Taylor C.W., Yeoman L.C. (1992). Inhibition of colon tumor cell growth by 8-chloro-cAMP is dependent upon its conversion to 8-chloro-adenosine. Anti-Cancer Drugs.

[19] Buettner R., Nguyen L.X.T., Morales C., Chen M.H., Wu X., Chen L.S., Hoang D.H., Hernandez Vargas S., Pullarkat V., Gandhi V. (2021). Targeting the metabolic vulnerability of acute myeloid leukemia blasts with a combination of venetoclax and 8-chloro-adenosine. J. Hematol. Oncol..

[20] Gandhi V., Ayres M., Halgren R.G., Krett N.L., Newman R.A., Rosen S.T. (2001). 8-chloro-cAMP and 8-chloro-adenosine act by the same mechanism in multiple myeloma cells. Cancer Res..

[21] Warner J.R. (1999). The economics of ribosome biosynthesis in yeast. Trends Biochem. Sci..

[22] Goodfellow S.J., Zomerdijk J.C. (2013). Basic mechanisms in RNA polymerase I transcription of the ribosomal RNA genes. Subcell Biochem..

[23] Nguyen D.Q., Hoang D.H., Nelson M., Nigam L., Nguyen V.T.T., Zhang L., Pham T.K.T., Ho H.D., Nguyen D.D.T., Lam T.Q. (2020). Requirement of GTP binding for TIF-90-regulated ribosomal RNA synthesis and oncogenic activities in human colon cancer cells. J. Cell. Physiol..

[24] Nguyen D.Q., Hoang D.H., Nguyen T.T.V., Ho H.D., Huynh V., Shin J.H., Ly Q.T., Thi Nguyen D.D., Ghoda L., Marcucci G. (2019). Ebp1 p48 promotes oncogenic activities in human colon cancer cells through regulation of TIF-90-mediated ribosomal RNA synthesis. J. Cell. Physiol..

[25] Nguyen L.X.T., Chan S.M., Ngo T.D., Raval A., Kim K.K., Majeti R., Mitchell B.S. (2014). Interaction of TIF-90 and filamin A in the regulation of rRNA synthesis in leukemic cells. Blood.

[26] Nguyen L.X.T., Lee Y., Urbani L., Utz P.J., Hamburger A.W., Sunwoo J.B., Mitchell B.S. (2015). Regulation of ribosomal RNA synthesis in T cells: Requirement for GTP and Ebp1. Blood.

[27] Nguyen L.X.T., Mitchell B.S. (2013). Akt activation enhances ribosomal RNA synthesis through casein kinase II and TIF-IA. Proc. Natl. Acad. Sci. USA.

[28] Nguyen L.X.T., Zhu L., Lee Y., Ta L., Mitchell B.S. (2016). Expression and Role of the ErbB3-Binding Protein 1 in Acute Myelogenous Leukemic Cells. Clin. Cancer Res. Off. J. Am. Assoc. Cancer Res..

[29] Nguyen L.X.T., Raval A., Garcia J.S., Mitchell B.S. (2015). Regulation of ribosomal gene expression in cancer. J. Cell. Physiol..

[30] Russell J., Zomerdijk J.C. (2005). RNA-polymerase-I-directed rDNA transcription, life and works. Trends Biochem. Sci..

[31] Puzio-Kuter A.M. (2011). The Role of p53 in Metabolic Regulation. Genes Cancer.

[32] Chapuis N., Poulain L., Birsen R., Tamburini J., Bouscary D. (2019). Rationale for Targeting Deregulated Metabolic Pathways as a Therapeutic Strategy in Acute Myeloid Leukemia. Front. Oncol..

[33] Sivandzade F., Bhalerao A., Cucullo L. (2019). Analysis of the Mitochondrial Membrane Potential Using the Cationic JC-1 Dye as a Sensitive Fluorescent Probe. Bio-Protocol.

[34] Karbowski M., Youle R.J. (2003). Dynamics of mitochondrial morphology in healthy cells and during apoptosis. Cell Death Differ..

[35] Konopleva M., Pollyea D.A., Potluri J., Chyla B., Hogdal L., Busman T., McKeegan E., Salem A.H., Zhu M., Ricker J.L. (2016). Efficacy and Biological Correlates of Response in a Phase II Study of Venetoclax Monotherapy in Patients with Acute Myelogenous Leukemia. Cancer Discov..

[36] Jordan C.T. (2019). Can we selectively target AML stem cells?. Best Pract. Res. Clin. Haematol..

[37] Stevens B.M., Jones C.L., Pollyea D.A., Culp-Hill R., D’Alessandro A., Winters A., Krug A., Abbott D., Goosman M., Pei S. (2020). Fatty acid metabolism underlies venetoclax resistance in acute myeloid leukemia stem cells. Nat. Cancer.

[38] Nguyen L.X.T., Troadec E., Kalvala A., Kumar B., Hoang D.H., Viola D., Zhang B., Nguyen D.Q., Aldoss I., Ghoda L. (2019). The Bcl-2 inhibitor venetoclax inhibits Nrf2 antioxidant pathway activation induced by hypomethylating agents in AML. J. Cell. Physiol..

[39] Leslie M. (2014). Central command. Science.

[40] Burger K., Muhl B., Harasim T., Rohrmoser M., Malamoussi A., Orban M., Kellner M., Gruber-Eber A., Kremmer E., Holzel M. (2010). Chemotherapeutic drugs inhibit ribosome biogenesis at various levels. J. Biol. Chem..

