# Supplementary material for: Synergy of Venetoclax and 8-Chloro-Adenosine in AML: The Interplay of rRNA Inhibition and Fatty Acid Metabolism
Source: Cancers (Basel). 2022 Mar 11;14(6):1446. doi: 10.3390/cancers14061446 (PMC8946614; doi:10.3390/cancers14061446)
Supplement: Supplementary file 1 [file cancers-14-01446-s001.zip › Supplementary File S1-Original Western Blots.pptx]

## Slide 1
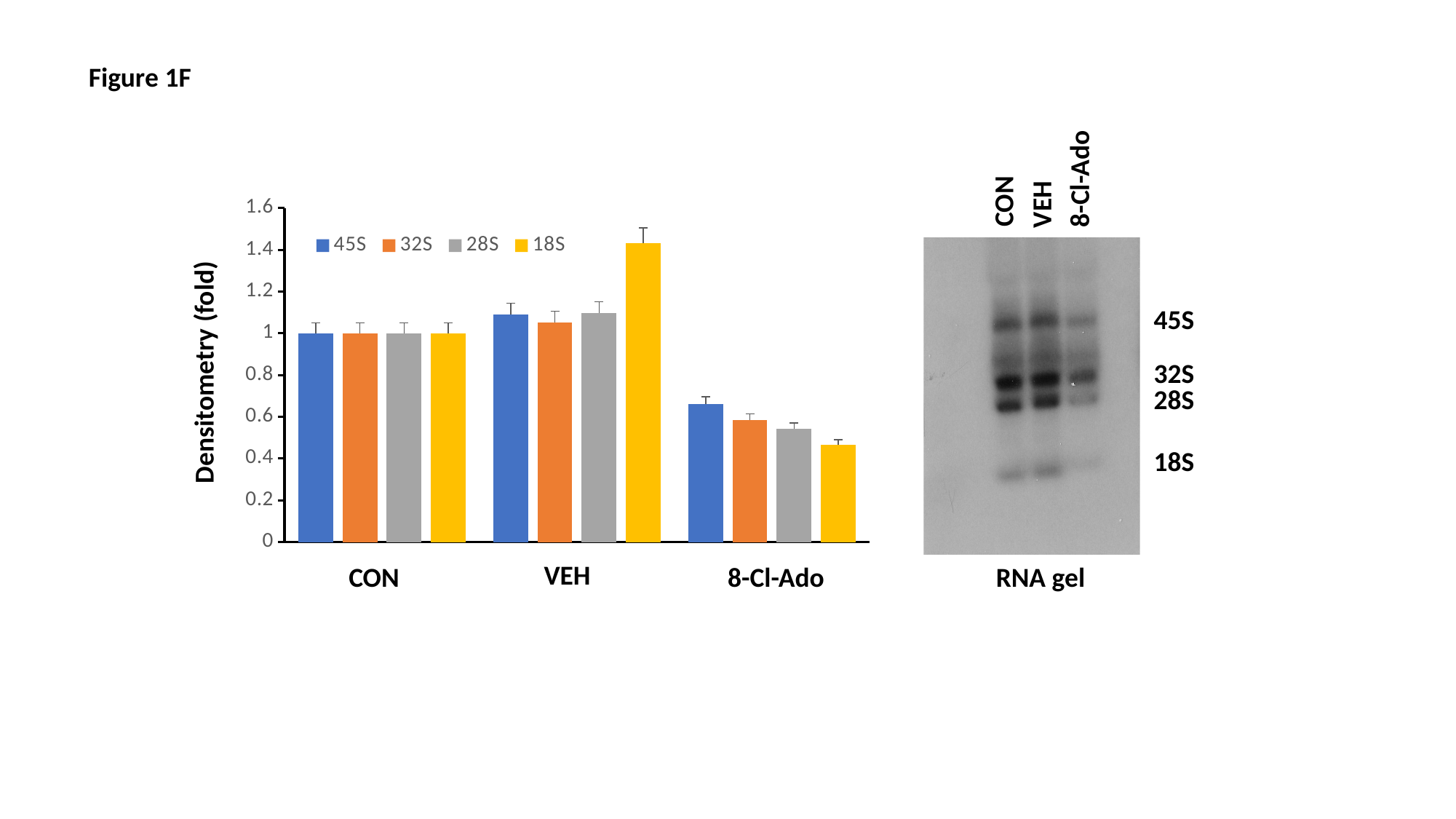

Figure 1F
8-Cl-Ado
CON
VEH
### Chart
| Category | | | | |
|---|---|---|---|---|45S
Densitometry (fold)
32S
28S
18S
VEH
CON
8-Cl-Ado
RNA gel

## Slide 2
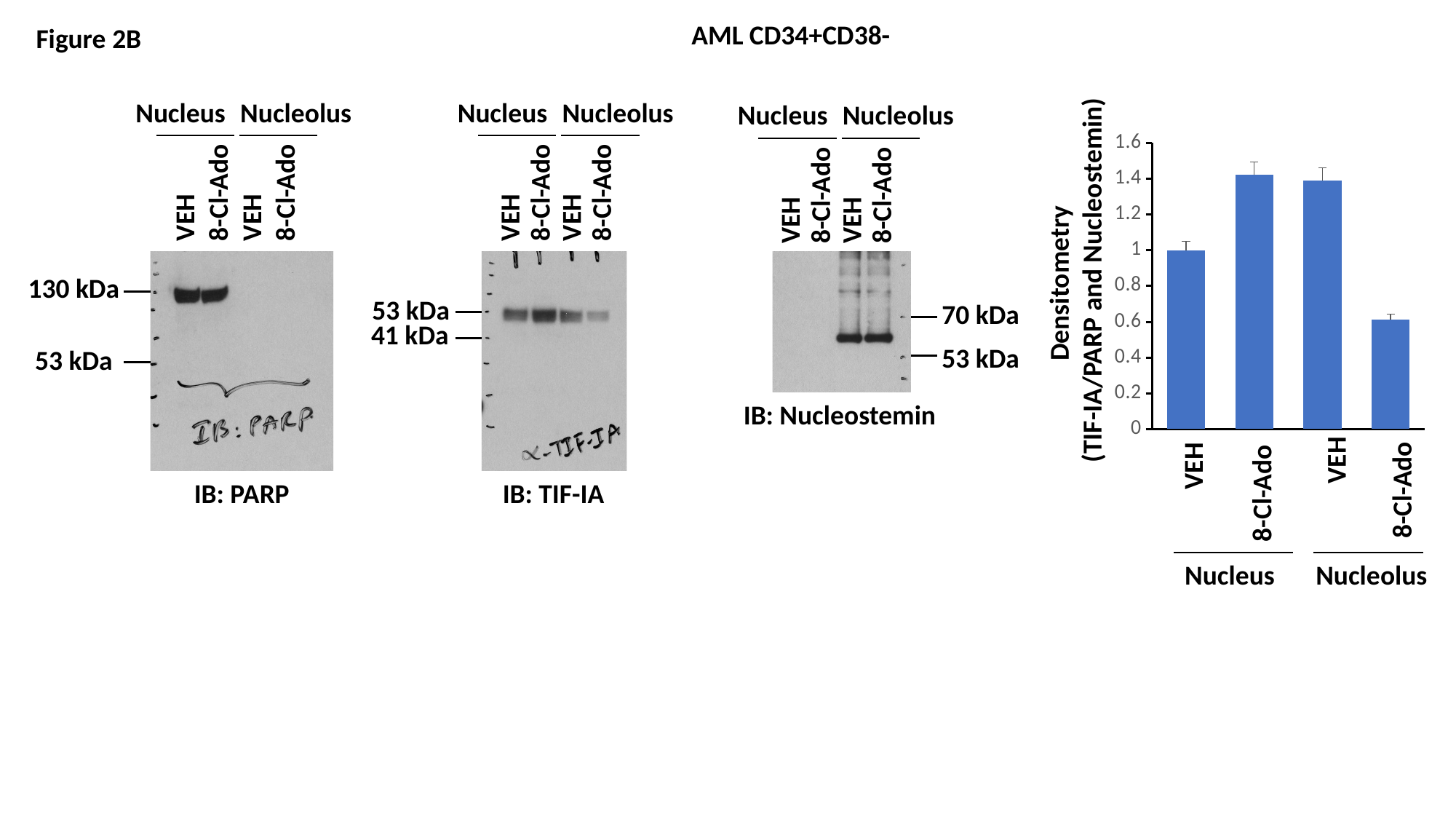

AML CD34+CD38-
Figure 2B
Nucleolus
Nucleolus
Nucleus
Nucleus
Nucleolus
Nucleus
### Chart
| Category | |
|---|---|8-Cl-Ado
8-Cl-Ado
8-Cl-Ado
8-Cl-Ado
8-Cl-Ado
8-Cl-Ado
VEH
VEH
VEH
VEH
VEH
VEH
Densitometry
(TIF-IA/PARP and Nucleostemin)
130 kDa
53 kDa
70 kDa
41 kDa
53 kDa
53 kDa
IB: Nucleostemin
VEH
VEH
8-Cl-Ado
8-Cl-Ado
IB: PARP
IB: TIF-IA
Nucleus
Nucleolus

## Slide 3
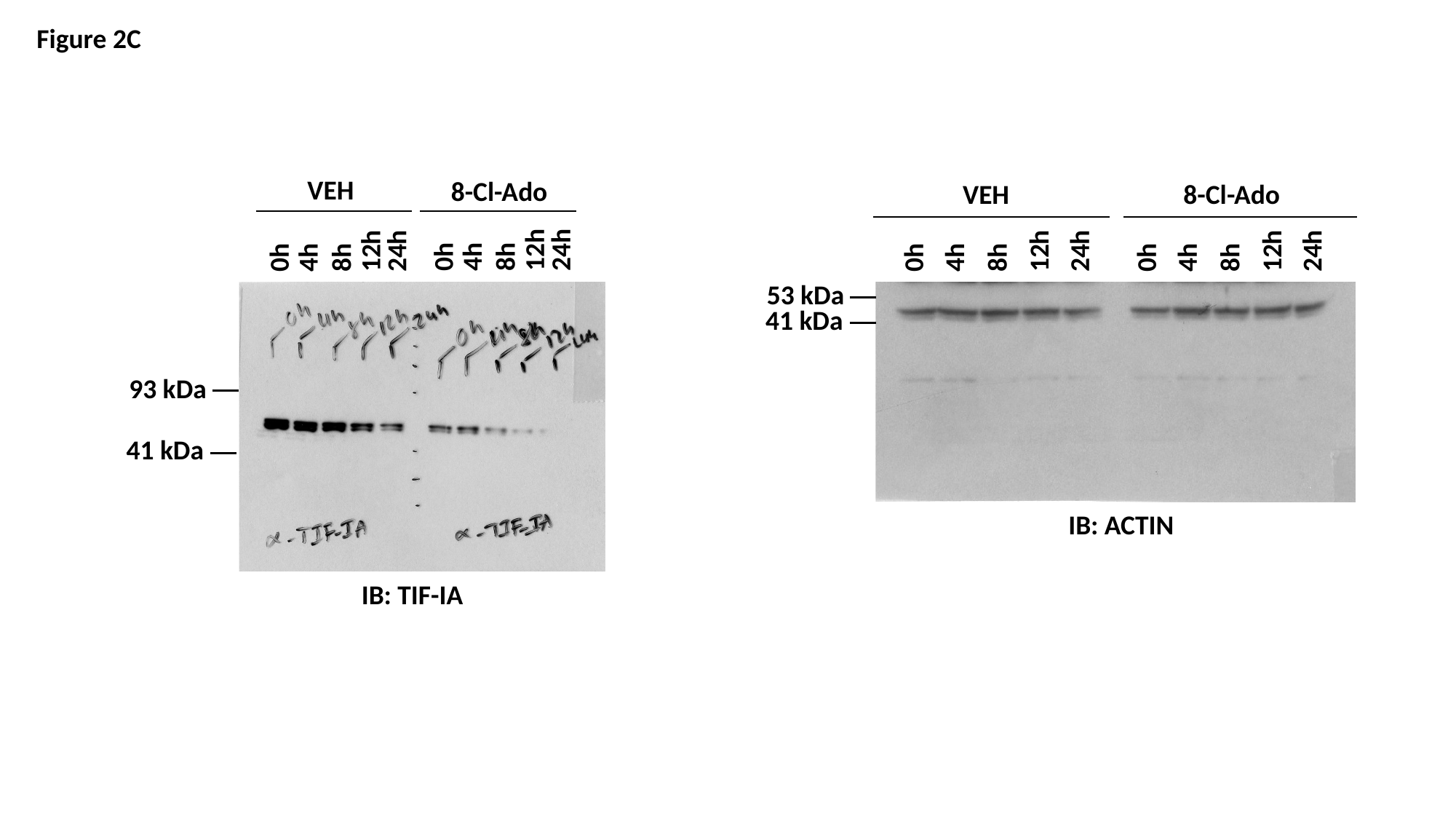

Figure 2C
VEH
8-Cl-Ado
VEH
8-Cl-Ado
0h
4h
8h
12h
24h
0h
4h
8h
12h
24h
0h
4h
8h
12h
24h
0h
4h
8h
12h
24h
53 kDa
41 kDa
93 kDa
41 kDa
IB: ACTIN
IB: TIF-IA

## Slide 4
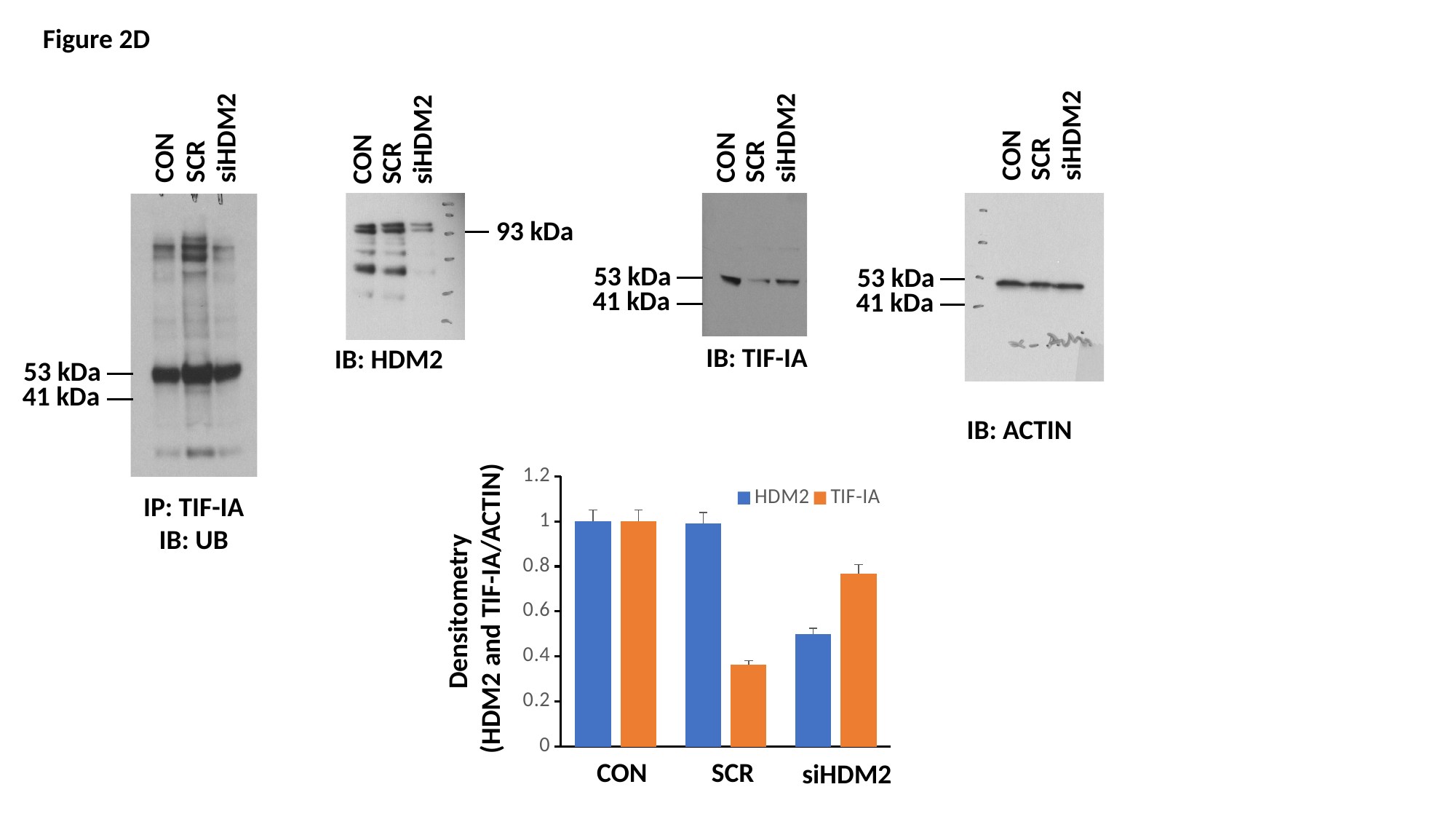

Figure 2D
siHDM2
siHDM2
siHDM2
siHDM2
SCR
CON
SCR
CON
SCR
CON
SCR
CON
93 kDa
53 kDa
53 kDa
41 kDa
41 kDa
IB: TIF-IA
IB: HDM2
53 kDa
41 kDa
IB: ACTIN
### Chart
| Category | | |
|---|---|---|IP: TIF-IA
IB: UB
Densitometry
(HDM2 and TIF-IA/ACTIN)
SCR
CON
siHDM2

## Slide 5
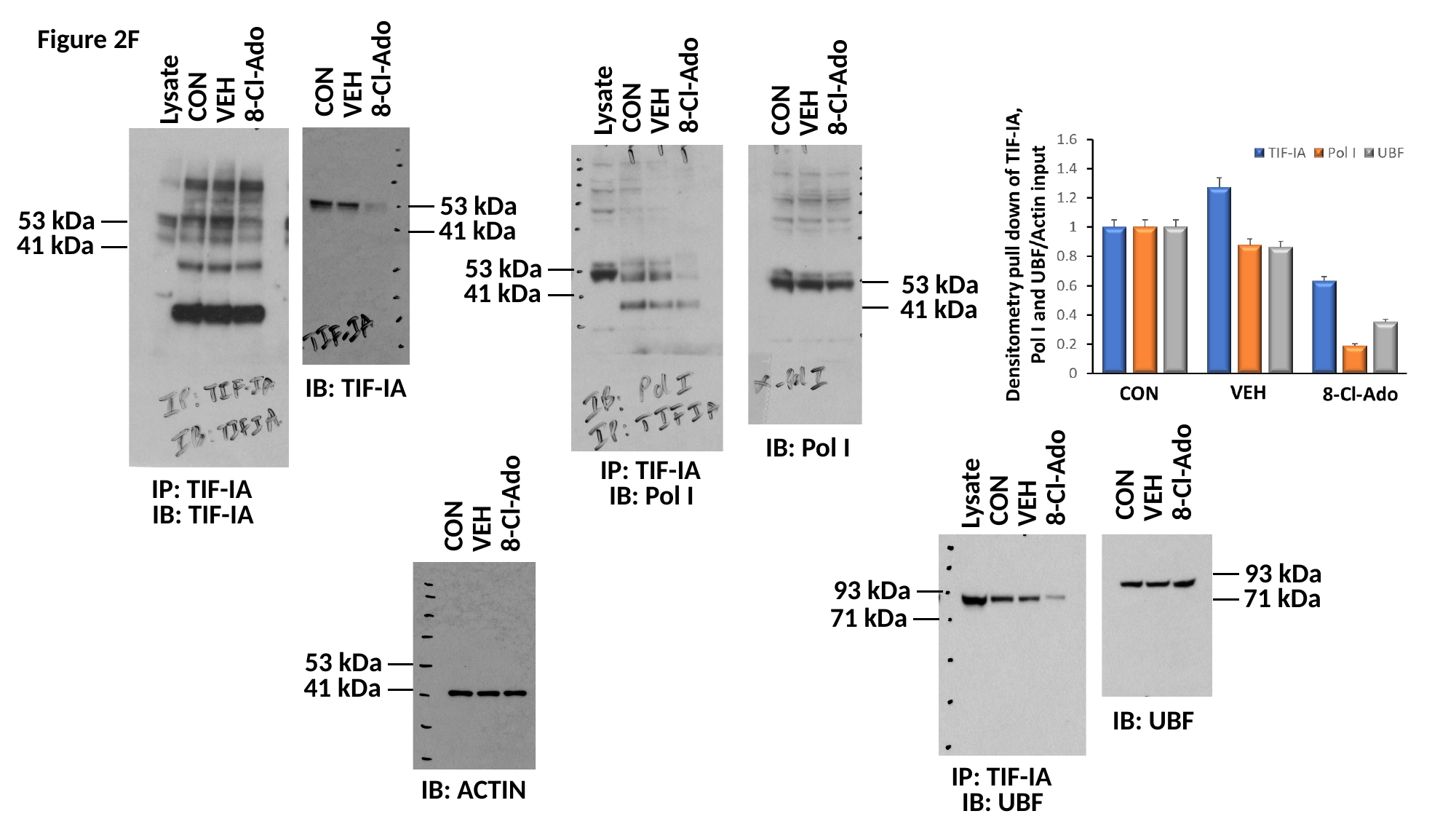

Figure 2F
8-Cl-Ado
8-Cl-Ado
Lysate
CON
VEH
CON
VEH
53 kDa
53 kDa
41 kDa
41 kDa
IB: Pol I
IP: TIF-IA
IB: Pol I
8-Cl-Ado
8-Cl-Ado
Lysate
CON
VEH
CON
VEH
53 kDa
53 kDa
41 kDa
41 kDa
IB: TIF-IA
8-Cl-Ado
CON
VEH
93 kDa
71 kDa
IB: UBF
8-Cl-Ado
Lysate
CON
VEH
93 kDa
71 kDa
IP: TIF-IA
IB: UBF
8-Cl-Ado
CON
VEH
53 kDa
41 kDa
IB: ACTIN
IP: TIF-IA
IB: TIF-IA

## Slide 6
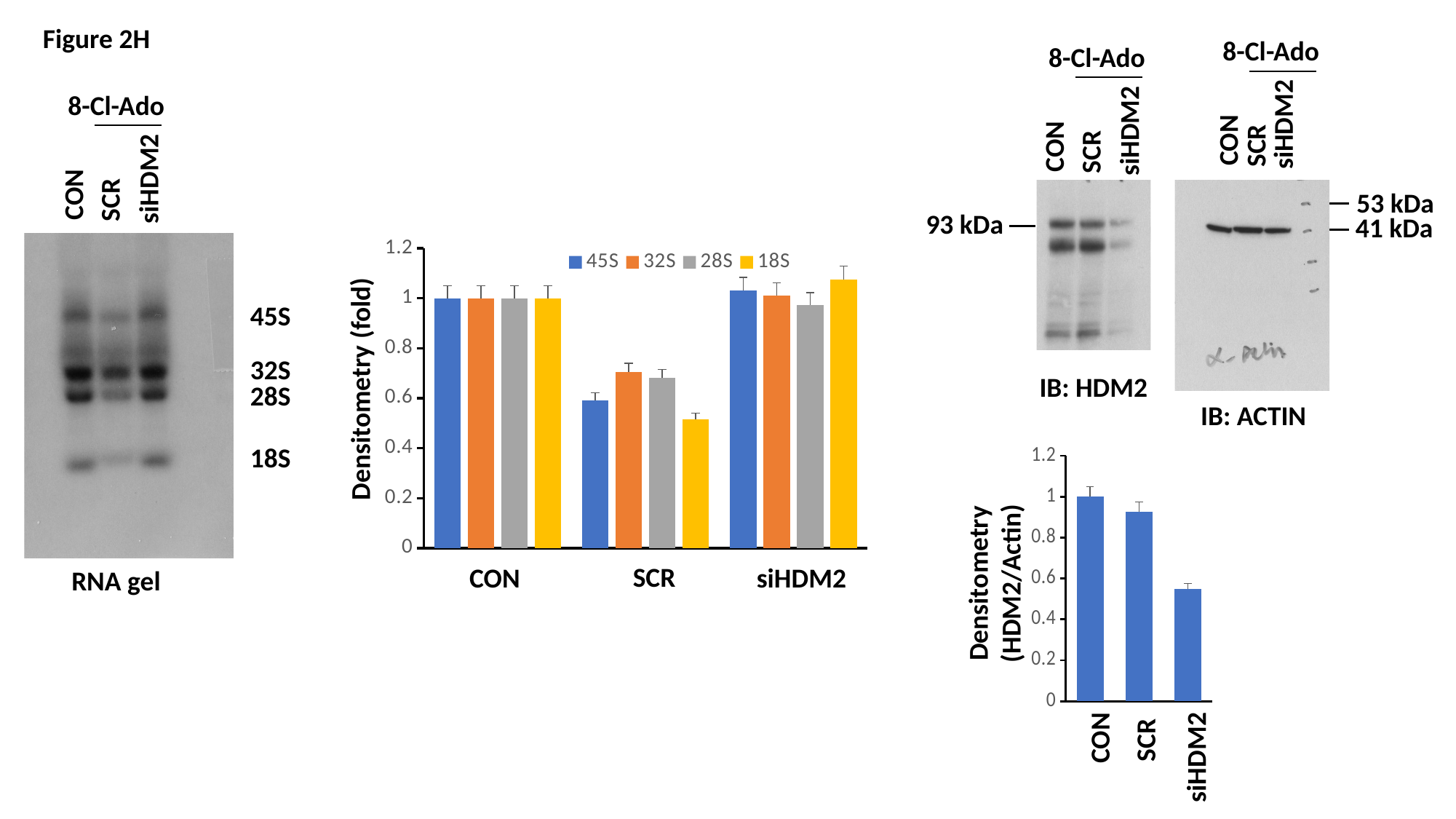

Figure 2H
8-Cl-Ado
8-Cl-Ado
8-Cl-Ado
siHDM2
siHDM2
CON
SCR
CON
SCR
siHDM2
CON
SCR
53 kDa
93 kDa
41 kDa
### Chart
| Category | | | | |
|---|---|---|---|---|45S
32S
IB: HDM2
Densitometry (fold)
28S
IB: ACTIN
18S
### Chart
| Category | |
|---|---|Densitometry (HDM2/Actin)
SCR
CON
siHDM2
RNA gel
SCR
CON
siHDM2

## Slide 7
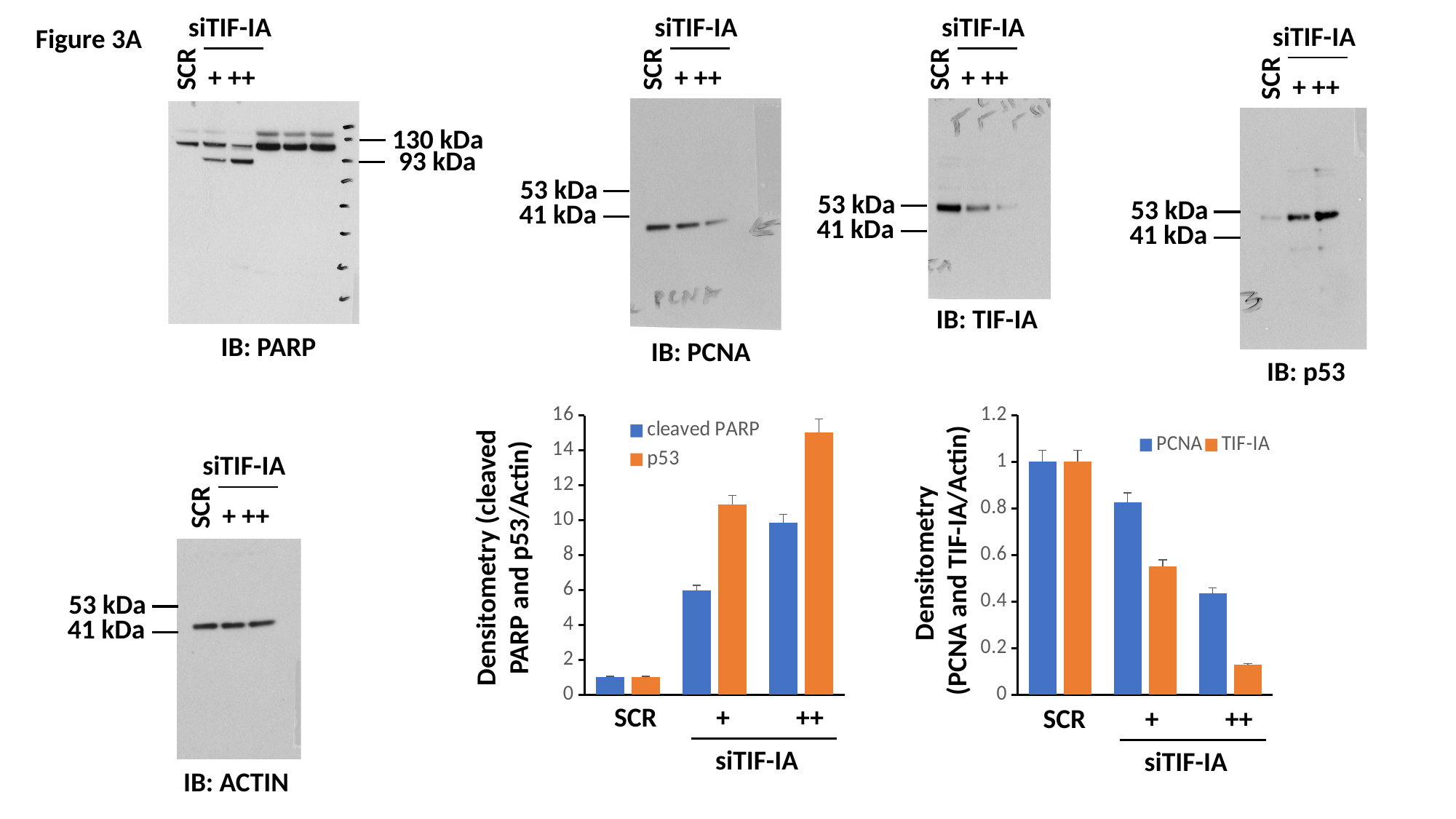

siTIF-IA
siTIF-IA
siTIF-IA
siTIF-IA
Figure 3A
SCR
SCR
SCR
SCR
+
++
+
++
+
++
+
++
130 kDa
93 kDa
53 kDa
53 kDa
53 kDa
41 kDa
41 kDa
41 kDa
IB: TIF-IA
IB: PARP
IB: PCNA
IB: p53
### Chart
| Category | | |
|---|---|---|
### Chart
| Category | | |
|---|---|---|siTIF-IA
SCR
+
++
Densitometry (cleaved PARP and p53/Actin)
Densitometry
(PCNA and TIF-IA/Actin)
53 kDa
41 kDa
SCR
+
++
SCR
+
++
siTIF-IA
siTIF-IA
IB: ACTIN

## Slide 8
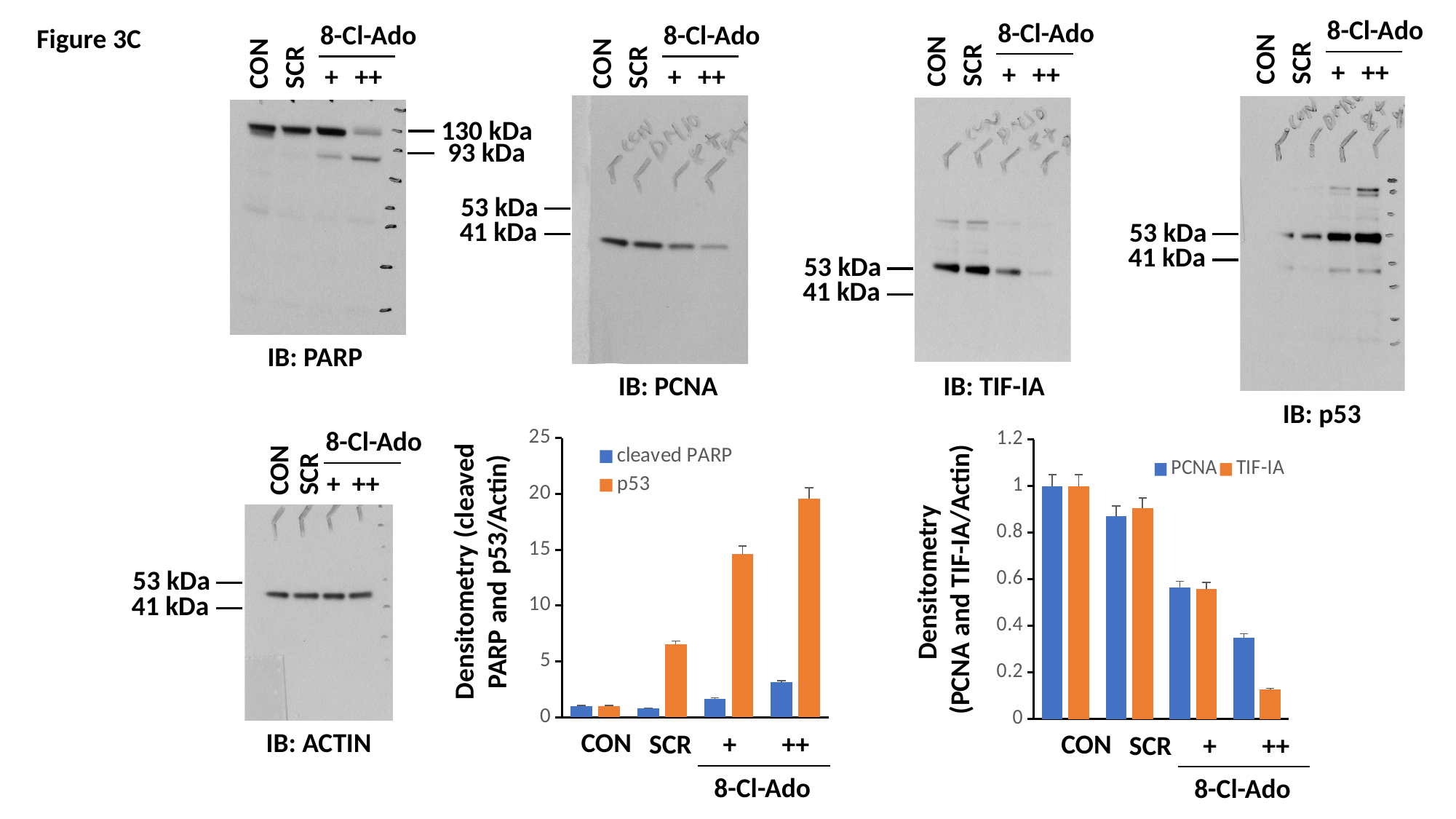

8-Cl-Ado
8-Cl-Ado
8-Cl-Ado
8-Cl-Ado
Figure 3C
CON
SCR
CON
SCR
CON
SCR
CON
SCR
+
++
+
++
+
++
+
++
130 kDa
93 kDa
53 kDa
41 kDa
53 kDa
41 kDa
53 kDa
41 kDa
IB: PARP
IB: PCNA
IB: TIF-IA
IB: p53
8-Cl-Ado
### Chart
| Category | | |
|---|---|---|
### Chart
| Category | | |
|---|---|---|CON
SCR
+
++
Densitometry (cleaved PARP and p53/Actin)
Densitometry
(PCNA and TIF-IA/Actin)
53 kDa
41 kDa
IB: ACTIN
CON
CON
SCR
+
++
SCR
+
++
8-Cl-Ado
8-Cl-Ado

## Slide 9
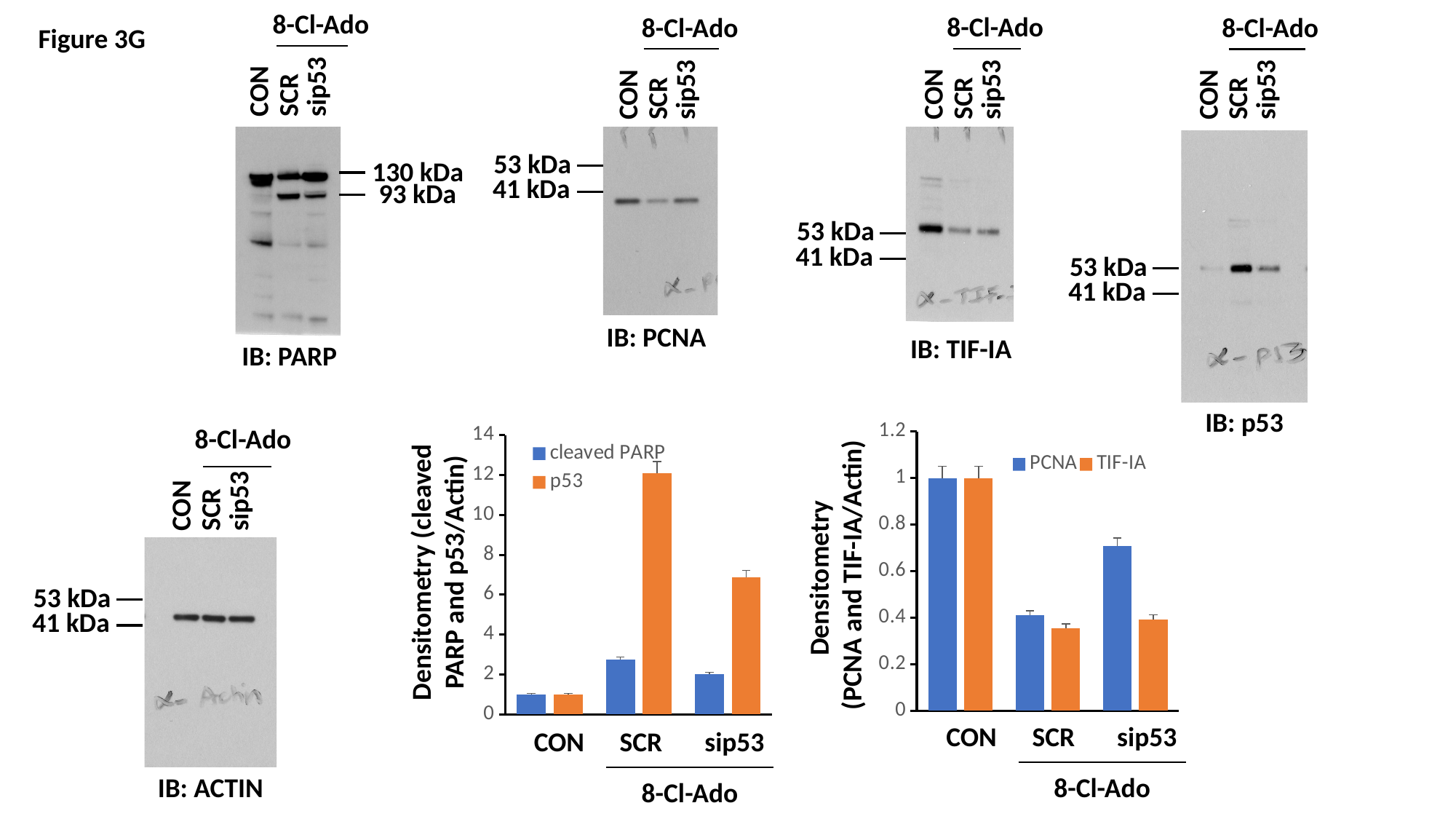

8-Cl-Ado
8-Cl-Ado
8-Cl-Ado
8-Cl-Ado
Figure 3G
sip53
CON
SCR
sip53
CON
SCR
sip53
CON
SCR
sip53
CON
SCR
53 kDa
130 kDa
41 kDa
93 kDa
53 kDa
41 kDa
53 kDa
41 kDa
IB: PCNA
IB: TIF-IA
IB: PARP
IB: p53
8-Cl-Ado
### Chart
| Category | | |
|---|---|---|
### Chart
| Category | | |
|---|---|---|sip53
CON
SCR
Densitometry (cleaved PARP and p53/Actin)
Densitometry
(PCNA and TIF-IA/Actin)
53 kDa
41 kDa
CON
SCR
sip53
CON
SCR
sip53
IB: ACTIN
8-Cl-Ado
8-Cl-Ado

## Slide 10
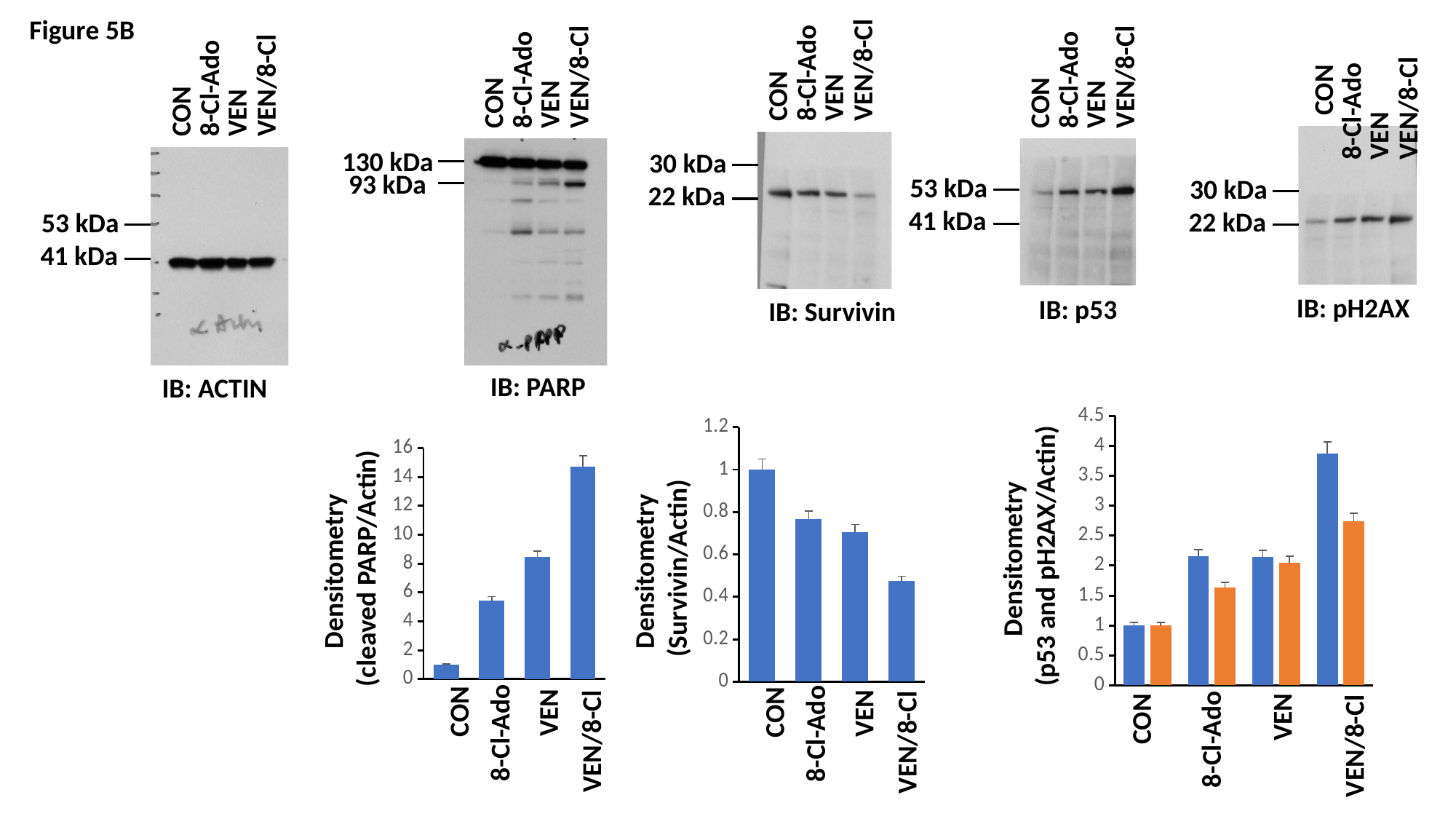

Figure 5B
VEN/8-Cl
VEN
8-Cl-Ado
VEN/8-Cl
VEN
8-Cl-Ado
VEN/8-Cl
VEN
8-Cl-Ado
VEN/8-Cl
VEN
8-Cl-Ado
CON
CON
CON
CON
CON
VEN/8-Cl
VEN
8-Cl-Ado
130 kDa
30 kDa
93 kDa
53 kDa
30 kDa
22 kDa
41 kDa
22 kDa
53 kDa
41 kDa
IB: pH2AX
IB: p53
IB: Survivin
IB: PARP
IB: ACTIN
### Chart
| Category | | |
|---|---|---|
### Chart
| Category | |
|---|---|
### Chart
| Category | |
|---|---|Densitometry
(p53 and pH2AX/Actin)
Densitometry
(cleaved PARP/Actin)
Densitometry
(Survivin/Actin)
CON
CON
CON
8-Cl-Ado
8-Cl-Ado
8-Cl-Ado
VEN
VEN/8-Cl
VEN
VEN/8-Cl
VEN/8-Cl
VEN

## Slide 11
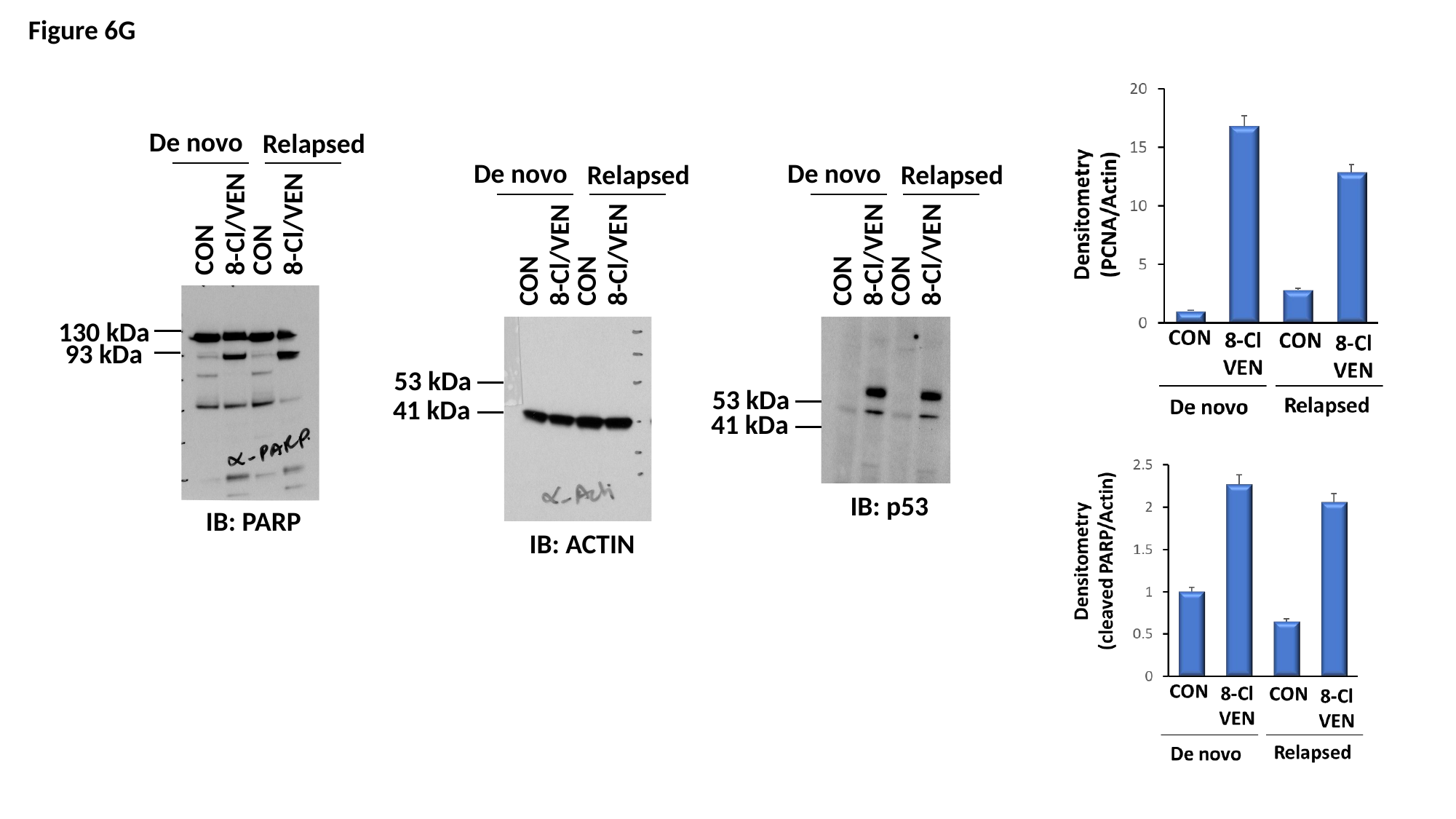

Figure 6G
De novo
Relapsed
8-Cl/VEN
8-Cl/VEN
CON
CON
130 kDa
93 kDa
IB: PARP
De novo
Relapsed
8-Cl/VEN
8-Cl/VEN
CON
CON
53 kDa
41 kDa
IB: ACTIN
De novo
Relapsed
8-Cl/VEN
8-Cl/VEN
CON
CON
53 kDa
41 kDa
IB: p53
