# Supplementary material for: Synergy of Venetoclax and 8-Chloro-Adenosine in AML: The Interplay of rRNA Inhibition and Fatty Acid Metabolism
Source: Cancers (Basel). 2022 Mar 11;14(6):1446. doi: 10.3390/cancers14061446 (PMC8946614; doi:10.3390/cancers14061446)
Supplement: Supplementary file 1 [file cancers-14-01446-s001.zip › Supplementary Tables and Figures.pdf]

SUPPLEMENTAL INFORMATION

1> **Supplemental Materials and Methods** (Page 2 to Page 5)

2> **Supplemental Tables** (Page 6 to Page 9)

- **+ Supplemental Table S1.** Patient characteristics of primary AML samples
- **+ Supplemental Table S2.** Target sequences of siRNAs
- **+ Supplemental Table S3.** Primer sequences used for ChiP and qPCR analysis
- **+ Supplemental Table S4.** List of antibodies used for ChiP, IF, IP and IB analysis

3> **Supplemental Figures S1 and S2 and Legends for Suppl. Figures S1 and S2** (Page 10)

4> **Quantification of original Western blot figures** (Page 11 to Page 21)

Supplemental Materials and Methods

Isolation of mononuclear cells from patient samples. Each patient specimen was transferred to a 50 mL conical tube and the volume was brought up to 25 mL using warm 1x Dulbecco's phosphate buffered saline (DPBS) with 2% FBS. The specimen was layered on the top of 20 mL Ficoll-Paque Plus in a 50 mL conical tube. Then, the tube was centrifuged at 300 g for 32 minutes without break. The layer containing PBMC and plasma was carefully transferred to a 50 mL conical tube and the volume was brought up to 50 mL with warm 1x DPBS. The tube was then centrifuged at 2400 rpm for 8 minutes. The supernatant was discarded, and the pellet was resuspended in 10 mL of warm DPBS (1x). Cell number and viability were determined, and the sample was frozen. $CD34^{+}CD38^{-}$ cells were then isolated using a magnetic bead selection protocol (Miltenyi Biotech, Germany).

Cell lines and chemicals. MV4-11 (RRID:CVCL_0064) and KG-1a (RRID:CVCL_1824) cells were purchased from the American Type Culture Collection (ATCC) and maintained in DMEM (Dulbecco's Modified Eagle Medium) supplemented with 10% FBS and 100 units of penicillin/streptomycin at 37°C with 5% CO_2. Human cell lines purchased from ATCC more than 6 months prior to submission of this manuscript and not frozen at an early passage were authenticated using ATCCs' human short tandem repeat (STR) DNA profiling authentication service. Morphology of cell lines was monitored routinely, and cell lines were routinely subjected to mycoplasma detection using a mycoplasma detection kit (Roche, Germany). Venetoclax was purchased from Selleckchem (Houston, Texas). 8-Chloro-adenosine was purchased from Tocris (Minneapolis, MN).

Synthetic small interfering RNA (siRNA) oligonucleotides. The siGENOME SMARTpool for siRNA of TIF-IA or HDM2 was purchased from Thermo Scientific (Lafayette, CO). Scrambled

control RNA (siSCR) was used as a control. The target sequences for siRNAs are shown in Supplemental Table 3.

Immunoprecipitation and immunoblotting analyses. Cells were washed and harvested in ice-cold PBS and subsequently lysed in buffer containing 1 mM phenylmethanesulfonylfluoride and 10 mM protease inhibitor cocktail. For immunoprecipitation, 500 µg of cell lysate was incubated with an anti-TIF-IA antibody overnight at 4 °C. 30 µl of Protein A/G agarose beads (Calbiochem) were added and the mixture was inverted for 2 hours at 4 °C. For immunoblotting, the immunoprecipitated complex or 30 µg of each cell lysate was separated on NuPAGE 4–12% gradient gels (Invitrogen) and immunocomplexes were visualized with enhanced chemiluminescence reagent (Thermo Scientific, Lafayette, CO). List of antibodies used for IP and IB analysis are shown on Supplemental Table S4.

DNA fragmentation analysis. Treated cells were lysed on ice for 60 min in 500 µL lysis buffer containing 0.02% SDS, 1% Nonidet P-40, and 0.2 mg/mL proteinase K in PBS. Genomic DNA was extracted using the phenol/chloroform method. The pellet was dissolved in 50 µL of TE buffer (supplemented with 10 mg/mL RNase) for 2 h at 37°C. A total of 10 µg of DNA was loaded on a 2% agarose gel and visualized under UV light.

FAO assay. Cultured cells were washed with HBSS and incubated with 200 µL of [^{3}H]-palmitic acid (1 mCi/ml, Perkin Elmer) bound to fatty-acid free albumin (100 µM; the ratio of palmitate:albumin is 2:1) and 1 mM l-cartinine. The complex was incubated for 2h at 37ºC. The supernatant was collected after incubation and added to a tube containing 200 µL of cold 10% trichloroacetic acid. The tubes were centrifuged 10 min at 3,000 g at 4ºC and aliquots of supernatants (350 µL) were removed, neutralized with 55 µL of 6N NaOH and applied to an ion exchange column loaded with Dowex 1X2 chloride form resin (Sigma Alrich). The radioactive

product was eluted with water. Flow-through was collected and radiation was quantified using liquid scintillation counting.

Seahorse assay. 40,000 cells in 200 uL cell culture medium were seeded in each well of a XF-96-well cell culture microplate and cultured overnight at 37ºC in 5% CO_2. As a negative control, three wells were kept devoid of cells and given only Seahorse media, which is comprised of basal XF media, 5.5 mM glucose, 1 mM sodium pyruvate, and 4 mM glutamine (additionally, the pH was adjusted to 7.4). Twelve hours prior to running a plate, the Seahorse sensor cartridge was incubated with Seahorse Calibrant solution according to the manufacturer's protocol, in a 37ºC, CO_2-free incubator. On the day of an assay, cells were washed and incubated with Seahorse media. The sensor cartridge was fitted onto the cell culture plate, which was then placed into a 37ºC, CO_2-free incubator for one hour. During the assay, which was run on the Seahorse XF96 Analyzer, the following inhibitors were injected sequentially, as is standard for the Cell Energy Test: oligomycin (1 mM), FCCP (0.5 mM).

Cellular Fractionation. The cells were collected and washed in PBS followed by fractionation into nucleolus and nucleus fractions using a subcellular fractionation kit (Thermo Scientific, Lafayette, CO). Briefly, the cells were vigorously vortexed in cytoplasmic extraction reagents and subsequently centrifuged to isolate the soluble cytoplasmic fraction. The remaining insoluble fraction, which contained nuclei, was suspended in nuclear extraction reagent and centrifuged to collect the nuclear fraction. All steps were performed at 4°C.

Measurement of mitochondrial membrane potential. Mitochondrial membrane potential was visualized in treated cell stained with JC-1 (Cat# T3168, ThermoFisher) using a confocal microscope (LSM880, Zeiss). Cells were collected, washed in ice-cold PBS and mounted on glass slides using a Cytocentrifuge (CytoSpin4, 600 rpm, 10 minutes). Cells were then washed with PBS, fixed in 4% paraformaldehyde for 15 minutes and permeabilized in 0.5% Triton X-100 for

15 minutes. Then the cells were stained with JC-1 dye for 1 hour at 37^0C. According to the manufacture, JC-1 dye exhibits potential-dependent accumulation in mitochondria, indicated by a green fluorescence emission at (~529 nm) for the monomeric form of the probe, which shifts to red (~590 nm) with a concentration-dependent formation of red fluorescent J-aggregates. Consequently, mitochondrial depolarization is indicated by a decrease in the red/green fluorescence intensity ratio (ThermoFisher).

Transmission electron microscopy. Cultured cells were fixed with 2.5% glutaraldehyte, 0.1M Cacodylate buffer ($Na(CH_3)_2AsO_2 \cdot 3H_2O$), pH7.2, at 4°C. Standard sample preparation for TEM was followed including post-fixation with osmium tetroxide, serial dehydration with ethanol, and embedment in Eponate. Ultra-thin sections (70 nm thick) were acquired by ultramicrotomy, post-stained, and examined on an FEI Tecnai 12 transmission electron microscope equipped with a Gatan OneView CMOS camera. TEM images were taken at nominal 11,000 x magnification.

Supplemental Table S1. Patient characteristics of primary AML samples

Sample Number	De Novo vs. Relapsed	Cytogenetic	% of Blasts cells
**For studies with de novo/relapsed AML samples:**			
AML001	De Novo	Normal	90
AML002	De Novo	46,XY, inv(16)(p13.1q22.1)[23]	60
AML003	De Novo	46,XY, i(21)(21)9q10)[20]	74
AML004	Relapsed	Stemline: 46,XY,add(22)(p11.20[4]<br>Sideline 1: 46,sl,t(3;13)(p25;q12)[11]<br>Sideline 2: 46,sdl1,add(21)(p11.2)[5]	78
AML005	Relapsed	Stemline: 46,XX,del(7)(q22q36)[9]<br>Sideline 1: 46,sl,t(1;16)(p13;p11.2)[2]<br>Sideline 2:46,sl,t(5;12)(q22;p13.2)[2]	78
AML006	Relapsed	46,XY,t(8;21)(q21.3;q22.12)[20]	85
AML007	Relapsed	Extremely complex	70

**For studies with pooled AML samples:**			
AML008	Induction failure	FLT3-ITD Pos., NPM1 Pos.	90
AML009	Relapsed	FLT3-ITD Pos., FLT3 D835 Neg., NPM1 Pos, C-Kit Neg.	95
AML010	De Novo	FLT3 Neg., NPM1 Pos.	90
AML011	Relapsed	CEBPA Pos., C-Kit Neg., FLT3 Neg., NPM1 Neg.	80
AML012	De Novo	FLT3-ITD Neg., NPM1 Neg.	74

Supplementary Table S2. The target sequences for siRNAs

No	Name	Target Sequence
1	siHDM2	**GCCAGUAUAUUAUGACUAA**<br>**GAUGAGAAGCAACAACAUA**<br>**AAAGUCUGUUGGUGCACAA**<br>**CCCUAGGAAUUUAGACAAC**
2	siTIF-IA	**ACACCAAGCUCCUUUGACA**<br>**CGAUGUAGAUGUUUCAGAU**<br>**CUAUGUAGAUGGUAAGGUU**<br>**AAACAAAGGAUCUAUAUCG**

Supplementary Table S3. Primer sequences used for ChIP and qPCR analysis

No	Primer name	Sequence
1	TIF-IA-F	5’ GTTCTTCTATCATGTACT 3’
2	TIF-IA-R	5’ TTGCAGGAAGATTATCAT 3’
3	ChIP-IGS-F	5’ TCGCCGACTCTCTCTTGACTTG 3’
4	ChIP-IGS-R	5’ TGGAGCACAGTGACACAACTATGG 3’
5	ChIP-promoter-F	5’ ATGGTGGCGTTTTTGGGG 3’
6	ChIP-promoter-R	5’ AGGCGGCTCAAGGCAGGAG 3’
7	5’ETS pre-RNA-F	5’ GAACGGTGGTGTGTCGTTC 3’
8	5’ETS pre-RNA-R	5’ GCGTCTCGTCTCGTCTCACT 3’
9	GAPDH-F	5’ CCCCTTCATTGACCTCAACTACAT 3’
10	GAPDH-R	5’ CGCTCCTGGAAGATGGTGA 3’

Supplementary Table S4. List of antibodies used for ChIP, IF, IP and IB analysis

No	Antibody name	Information
1	Anti-p53 antibody	Clone# DO-1, Cat# sc-126, Santa Cruz
2	Anti-p-H2AX antibody	Cat# ab81299, Abcam
3	Anti-Survivin antibody	Clone# 71G4B7, Cat# 2808, Cell Signaling
4	Anti-TIF-IA antibody	Cat# SAB4502266, Sigma
5	Anti-PCNA antibody	Cat# sc-56, Santa Cruz
6	Anti-UBF antibody	Clone# F-9, Cat# sc-13125, Santa Cruz
7	Anti-ACTIN antibody	Clone# C4, Cat# sc-47778, Santa Cruz
8	Anti-PARP antibody	Cat# 9542, Cell Signaling
9	Anti-Pol-I antibody	Cat# HPA022416, Sigma
10	Anti-Mdm2 antibody	Cat# sc-813, Santa Cruz
11	Anti-Ub antibody	Cat# 07-375, Millipore
12	Anti-Nucleostemin antibody	Cat# sc-46215, Santa Cruz

Supplemental Figure S1: Effects of 8-Cl-Ado plus VEN on mitochondria metabolism.

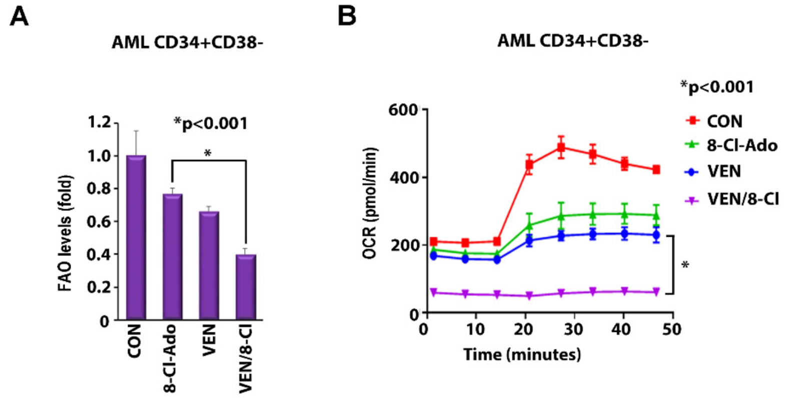

Supplemental Figure S1: Effects of 8-Cl-Ado plus VEN on mitochondria metabolism. LSC-enriched blast cells were treated with 500 nM 8-Cl-Ado, 10 nM VEN, or both, for 24h. (A) Levels of FAO were measured by the oxidation rate of ^{3}H-palmitic acid. (B) Levels of oxidative consumption rate (OCR) were measured by seahorse cell energy testing assay.

Supplemental Figure S2: Effect of 8-Cl-Ado plus VEN on mitochondrial membrane potential.

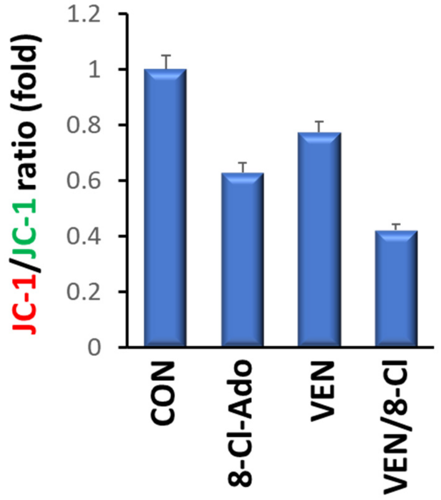

Supplemental Figure S2: Effect of 8-Cl-Ado plus VEN on mitochondrial membrane potential. LSC-enriched blast cells were treated with 500 nM 8-Cl-Ado, 10 nM VEN, or both, for 24h. The effect of drug-treatment on mitochondrial membrane potential was quantified by measuring the ratio of JC-1 aggregates (red fluorescence, sign of healthy mitochondria) to JC-1 monomers (green fluorescence, sign of unhealthy mitochondria).
